# NADase CD38 is a key determinant of ovarian aging

**DOI:** 10.1038/s43587-023-00532-9

**Published:** 2023-12-21

**Authors:** Qingling Yang, Wenhui Chen, Luping Cong, Mengchen Wang, Hui Li, Huan Wang, Xiaoyan Luo, Jing Zhu, Xinxin Zeng, Zhenye Zhu, Yining Xu, Min Lei, Yanqing Zhao, Chenlu Wei, Yingpu Sun

**Affiliations:** 1 Center for Reproductive Medicine, The First Affiliated Hospital of Zhengzhou University, Zhengzhou, China; 2 Henan Key Laboratory of Reproduction and Genetics, The First Affiliated Hospital of Zhengzhou University, Zhengzhou, China; 3 Henan Provincial Obstetrical and Gynecological Diseases (Reproductive Medicine) Clinical Research Center, The First Affiliated Hospital of Zhengzhou University, Zhengzhou, China

**Keywords:** Reproductive disorders, Ageing

## Abstract

The ovary ages earlier than most other tissues, yet the underlying mechanisms remain elusive. Here a comprehensive analysis of transcriptomic landscapes in different organs in young and middle-aged mice revealed that the ovaries showed earlier expression of age-associated genes, identifying increased NADase CD38 expression and decreased NAD^+^ levels in the ovary of middle-aged mice. Bulk and single-cell RNA sequencing revealed that CD38 deletion mitigated ovarian aging, preserving fertility and follicle reserve in aged mice by countering age-related gene expression changes and intercellular communication alterations. Mechanistically, the earlier onset of inflammation induced higher expression levels of CD38 and decreased NAD^+^ levels in the ovary, thereby accelerating ovarian aging. Consistently, pharmacological inhibition of CD38 enhanced fertility in middle-aged mice. Our findings revealed the mechanisms underlying the earlier aging of the ovary relative to other organs, providing a potential therapeutic target for ameliorating age-related female infertility.

## Abstract (web summary)

Multiple lines of research show that NAD^+^ has an important role in ovarian aging; however, the role of NAD^+^ consumption during ovarian aging is incompletely understood. Here the authors study the role of the NADase CD38 to show that CD38 expression increases and NAD^+^ levels decrease with age in mice, and that CD38 deletion ameliorates ovarian aging.

## Main

Ovarian functional lifespan is determined by the size of the nonrenewable ovarian follicle reserve at birth, as well as the rate at which this endowment is depleted, ending with menopause^1^. Modern trends of delayed childbearing lead to elevated infertility, miscarriages and birth defects in the offspring^2^. Consequently, there is increased demand for the treatment of age-induced female infertility due to decreased oocyte quantity and quality^3^. Thus, comprehending physiological and molecular mechanisms of ovarian aging is crucial for effective infertility treatment and healthcare management.

The ovary ages early in females, with menopause around 50–52 years, much shorter than modern life expectancy of up to 90 years^4,5^. Ovarian aging, marked by fertility decline and hormonal changes, impacts overall health due to the influence of ovarian hormones on various tissues, such as reproductive, bone, brain and skin tissues^6,7^. Ovarian aging is associated with an increased risk of diabetes, heart disease, cancer and other conditions^6,8–11^. Transplanting young mouse ovaries into older mice extends lifespan^12^, improving cognitive, immune and renal functions^13^. Nevertheless, the molecular basis for the accelerated onset of ovarian aging compared to other organs remains unclear.

The ovarian follicle consists of an oocyte surrounded by granulosa and theca cells^14^. Oocyte–granulosa–theca cell interactions regulate follicular growth and atresia^15^. Dysfunction of follicles leads to premature ovarian insufficiency^16–19^. Although the detailed mechanisms underlying ovarian aging are unknown, studies have found that ovarian aging leads to the accumulation of reactive oxygen species (ROS) and mitochondrial dysfunction in oocytes, resulting in aneuploidy associated with decreased embryonic development^20–23^. Accumulating evidence has revealed that oxidative stress is one of the key factors inducing oocyte and granulosa cell apoptosis in mice^24^. Monkey studies have also demonstrated that oxidative damage is a crucial factor in ovarian aging^25^. In addition, inflammation genes play an important role in maintaining ovary function, and genetic deletion of inflammation-related genes, such as those encoding tumor necrosis factor (TNF), interleukin-1 alpha (IL-1α) and NLR family pyrin domain-containing 3 (NLRP3), delayed ovarian aging by inhibiting follicular atresia and improving oocyte quality^26–29^.

In the present study, we analyzed the transcriptome of the ovaries, together with those of the liver, muscle, brain, heart, kidney and lung from young (2-month-old) and middle-aged (8-month-old) mice. We found an earlier onset of inflammation-induced CD38 activation and decreased NAD^+^ levels in the ovary compared with other organs in middle-aged mice, resulting in follicle depletion and decreased oocyte quality during aging. Bulk RNA sequencing (RNA-seq) and single-cell RNA sequencing (scRNA-seq) revealed that genetic deletion of CD38 rescued age-related changes in gene expression. These changes involve genes associated with pathways such as the senescence-associated secretory phenotype (SASP), DNA repair, inflammation, core regulatory transcription factors (TFs) and cell–cell communication networks, ultimately resulting in higher female fertility. Furthermore, pharmacological inhibition of CD38 increased ovarian NAD^+^ levels and reduced inflammation, leading to improved oocyte quality. Overall, this study provides a comprehensive molecular understanding of ovarian senescence earlier than in other organs, offering potential clinical treatment strategies for ovarian aging.

## Results

### Ovary ages earlier than several other organs in middle-aged mice

**Fig. 1:**
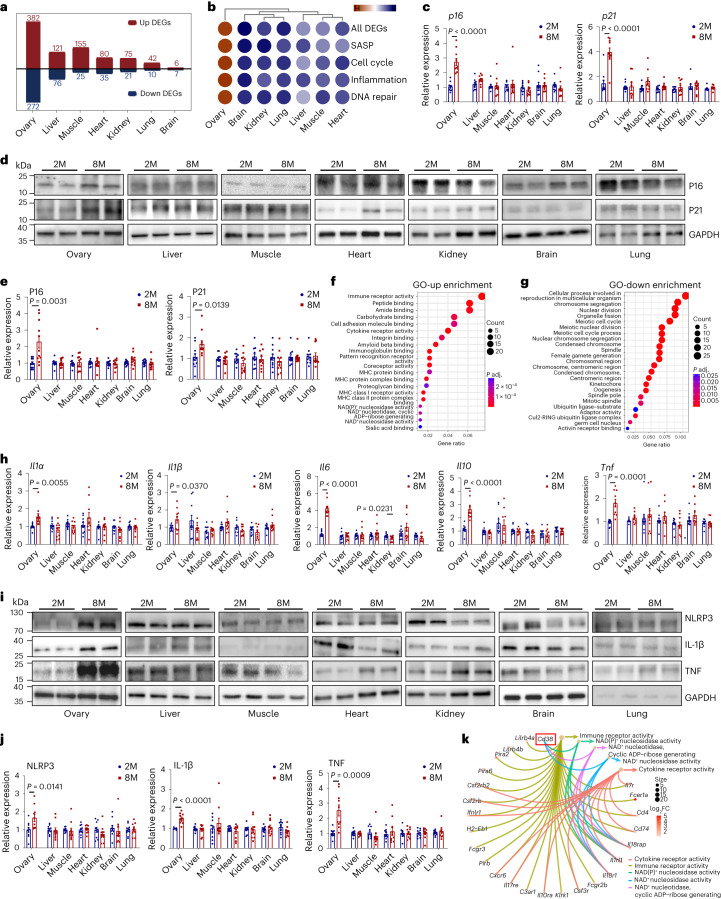
Earlier onset of senescence in the ovary than in other organs in middle-aged (8-month-old) compared with young (2-month-old) mice. **a**, Transcriptomic analysis of the upregulated (up) and downregulated (down) DEGs in the ovary, liver, muscle, heart, kidney, brain and lung tissues between 2-month-old and 8-month-old mice. Genes with |log_2_fold change (FC)| > 1 and adjusted *P* value (by the Benjamini–Hochberg method) < 0.05 were considered DEGs (*n* = 5 ovaries and *n* = 3 other tissues for each group). **b**, Dot plot of aging-related DEGs in the ‘SASP’, ‘cell cycle’, ‘inflammation’ and ‘DNA repair’ pathways, showing increased expression in the ovary, but not in other tissues tested, in middle-aged mice. **c**, Increased transcription of the senescence markers *p16* and *p21* in the ovaries but not in other organs from 8-month-old mice by using real-time RT–PCR (*n* = 9 or 10 mice for each group). M, months. **d**, Immunoblotting for the senescence markers P16 and P21 in the ovaries and other organs from 2-month-old and 8-month-old mice (*n* = 11 mice for each group). **e**, Quantification of P16 and P21 protein levels compared with GAPDH levels in different tissues (*n* = 11 mice for each group). **f**,**g**, GO analysis identified upregulated (**f**) and downregulated (**g**) genes in different pathways between young and middle-aged mice. *P* values were calculated by Fisher’s exact test and adjusted by the Benjamini–Hochberg method. **h**, Inflammation-related mRNA expression in the ovary, liver, muscle, heart, kidney, brain and lung from 2-month-old and 8-month-old mice (*n* = 9 or 10 mice for each group). **i**, Immunoblotting for inflammation-related protein expression in the ovary, liver, muscle, heart, brain, kidney and lung from 2-month-old and 8-month-old mice. **j**, The relative expression of each protein was calculated as a ratio to GAPDH levels (*n* = 10 mice for each group). **k**, Cnetplot showing the top five pathways from the GO ‘molecular function’ category and related genes with *Cd38* highlighted. Data are presented as the mean ± s.e.m. *P* value was determined by the unpaired, two-tailed *t*-test. MHC, major histocompatibility complex; NLRP, NOD-containing, LRR-containing and pyrin domain-containing protein. Source data

**Extended Data Fig. 1:**
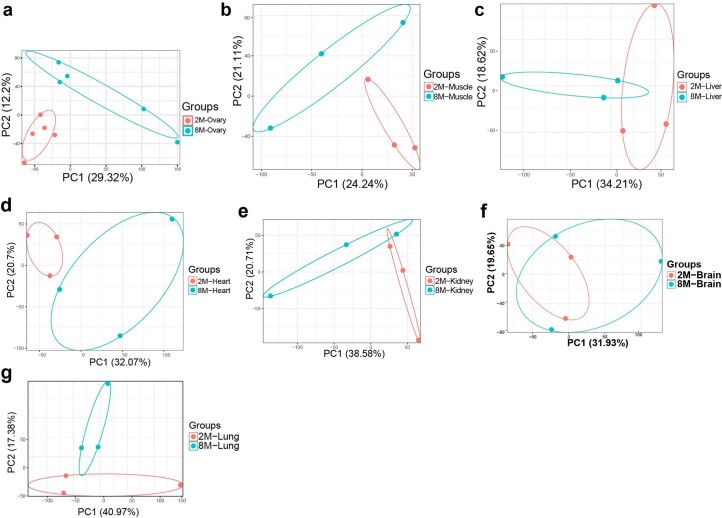
Transcriptome data from different organs in 2-month and 8-month-old mice. **a–g**. Principal component analysis (PCA) of RNA transcriptomes in the ovary (a), muscle (b), liver (c), heart (d), kidney (e), brain (f), and lung (g). Source data

Although the ovary ages earlier than most other organs in the body^30,31^, a systematic molecular comparison among tissues is lacking. We performed comparative transcriptomic analysis of the ovary, heart, kidney, brain, lung, liver and muscle by using bulk RNA-seq from young and middle-aged female mice at 2 and 8 months of age, respectively. As shown in Fig. 1a, middle-aged ovaries had more differentially expressed genes (DEGs), followed by liver, muscle, heart, kidney, lung and brain (Supplementary Table 1 and Extended Data Fig. 1a–g). By analyzing gene transcription in senescence-related pathways, including ‘SASP’, ‘cell cycle’, ‘inflammation’ and ‘DNA repair’, we found that a larger number of transcripts in these pathways had altered expression in the ovary relative to other organs when comparing 8-month-old with 2-month-old mice (Fig. 1b). Increased expression of two senescence markers, namely, p16 and p21, was confirmed by using real-time PCR with reverse transcription (RT–PCR) and western blotting in middle-aged ovaries, but not in other tissues, when comparing 2-month-old and 8-month-old mice (Fig. 1c–e). These results suggest that ovarian senescence occurred earlier than that of most other organs in mice.

### Inflammation activation was earlier in the middle-aged ovary

To unveil mechanisms underlying ovarian aging, we performed Gene Ontology (GO) enrichment analysis on DEGs from the RNA-seq data between young (2-month-old) and middle-aged (8-month-old) ovaries. Upregulated DEGs in middle-aged ovaries were enriched in inflammation pathways encompassing ‘immune response activity’ and ‘immunoglobulin binding’ (Fig. 1f). In contrast, downregulated genes showed enrichment in pathways like ‘female gamete generation’, ‘meiotic cell cycle process’ and ‘oogenesis’ (Fig. 1g), indicating decreased ovarian function in middle-aged mice. Real-time RT–PCR and western blotting showed that ovarian gene transcripts for diverse inflammation factors were increased in middle-aged versus young mice, whereas no differences were observed in the other tissues tested (Fig. 1h–j).

The top five pathway-related genes from the GO ‘molecular function’ category are shown in Fig. 1k. Of note, the NAD^+^ consumption enzyme CD38, which is involved in three different pathways (NAD^+^ nucleosidase activity, NAD(P)^+^ nucleosidase activity and NAD^+^ nucleosidase), was upregulated in the ovaries of middle-aged mice. Considering that the decline in the NAD^+^ level induced by the increase in CD38 expression plays an important role in the liver, skeletal muscle and adipose tissue in aged mice^32^, we focused on the role of CD38 in the ovary.

### Increased CD38 expression in middle-aged ovaries

**Fig. 2:**
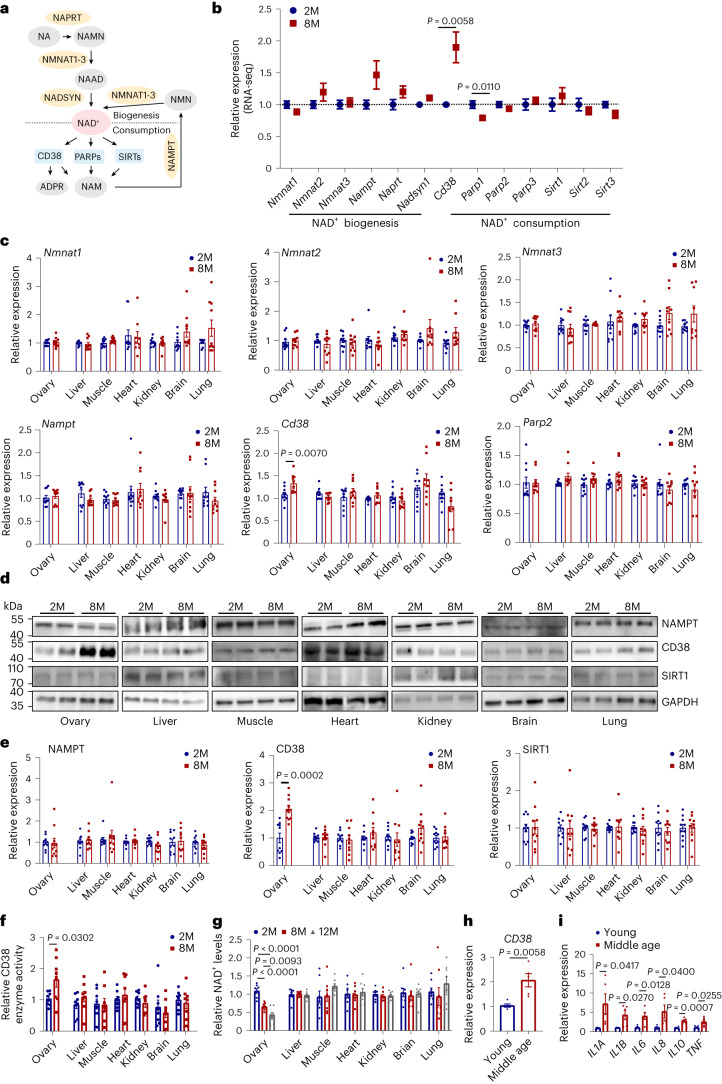
Increased expression of CD38 and decreased NAD^+^ levels in ovaries but not in other tissues in middle-aged mice. **a**, Diagram showing NAD^+^ biogenesis and consumption enzymes. **b**, RNA-seq data showing the gene transcription levels for NAD^+^ biogenesis and consumption enzymes in 2-month-old and 8-month-old ovaries. **c**, Gene transcript analysis by real-time RT‒PCR for some of the key enzymes in the NAD^+^ biogenesis pathways (*Nmnat1*, *Nmnat2*, *Nmnat3* and *Nampt*) and consumption pathways (*Cd38* and *Parp2*) in different organs in 2-month-old and 8-month-old mice (*n* = 9 or 10 mice for each group). **d**, Immunoblotting for protein expression of the rate-limiting enzyme in the NAD^+^ biogenesis (NAMPT) and consumption pathways (CD38 and SIRT1) in different organs of 2-month-old and 8-month-old mice. **e**, Quantification of the relative expression of each protein calculated as a ratio to GAPDH levels in different tissues from 2-month-old and 8-month-old mice (*n* = 10 mice for each group). **f**, CD38 enzyme activities in different organs during aging were detected by an ELISA (*n* = 8 mice for each group). **g**, NAD^+^ levels in different tissues during aging in mice (*n* = 8 mice for each group). **h**, CD38 transcript levels in isolated cells from follicle fluid using flow cytometry from young (20–25 years old) and middle-aged (>35 years old) individuals (*n* = 5 for each gene). **i**, Inflammation-associated gene expression was determined by real-time RT‒PCR using isolated cells from young and middle-aged individuals (*n* = 5 for each gene). Data are presented as the mean ± s.e.m. *P* value was determined by the unpaired two-tailed *t*-test between the two groups. Source data

NAD^+^ metabolism plays an important role during aging in mammals^33–37^. The transcription levels of key enzymes responsible for NAD^+^ biogenesis and consumption (Fig. 2a) were analyzed in ovaries using transcriptomic sequencing. As shown in Fig. 2b, the expression levels of NAD^+^ biogenesis genes were not changed in the middle-aged ovary. Although no changes in most NAD^+^ consumption genes were found, increased transcription of *Cd38* was evident (Fig. 2b). To confirm this finding, we further determined the expression of some key enzymes for NAD^+^ biogenesis and consumption in different tissues of middle-aged mice using real-time RT‒PCR and immunoblotting. The rate-limiting NAD^+^ biogenesis enzyme NAMPT’s transcription and protein expression remained unchanged in both the middle-aged ovary and other tested tissues (Fig. 2c–e). Furthermore, the key genes (*Nmnat1*, *Nmnat2* and *Nmnat3*) responsible for NAD^+^ biogenesis exhibited consistent expression across all tissues tested, indicating unaltered NAD^+^ biogenesis in middle-aged tissues. Conversely, the NADase CD38 exhibited a notable increase in expression in the middle-aged ovary (Fig. 2c–e), whereas other enzymes (SIRT1 and PARP2) in NAD^+^ consumption pathways remained unchanged across all tissues (Fig. 2c–e). Enzyme activity assessment revealed an augmented CD38 activity specifically in the ovaries of middle-aged mice compared to young mice, while no such change was observed in other tissues (Fig. 2f). Consistent with the increased ovarian expression of CD38, we found a marked decrease in ovarian NAD^+^ levels in middle-aged mice but nonotable differences in other tissues (Fig. 2g). To determine if the increased expression of CD38 observed in middle-aged mice is also present in the human female reproductive system, follicular fluids were collected from young (20–25 years old) and middle-aged (>35 years old) individuals with blocked fallopian tubes. Decreased ovarian reserve was observed in middle-aged individuals, as demonstrated by the lower anti-Mullerian hormone (AMH) levels, fewer antral follicles and the lower number of retrieved oocytes compared with those in young individuals (Supplementary Table 2). CD38-positive cells were isolated using flow cytometry (Supplementary Fig. 1). Interestingly, human *CD38* mRNA expression was higher in purified cells from middle-aged individuals compared to young individuals (Fig. 2h). Real-time RT–PCR analysis also revealed elevated transcript levels of age-related inflammation factors, including *IL1A*, *IL1B*, *IL6*, *IL8*, *IL10* and *TNF*, in CD38-positive cells from middle-aged individuals relative to younger individuals (Fig. 2i). These results suggest that increased expression of CD38 in middle-aged ovaries is a common feature in mammals, indicating that the earlier onset of increased CD38 expression and declining NAD^+^ levels may underlie the earlier senescence in the ovary relative to other organs.

### Deletion of CD38 improved the fecundity in female mice

**Fig. 3:**
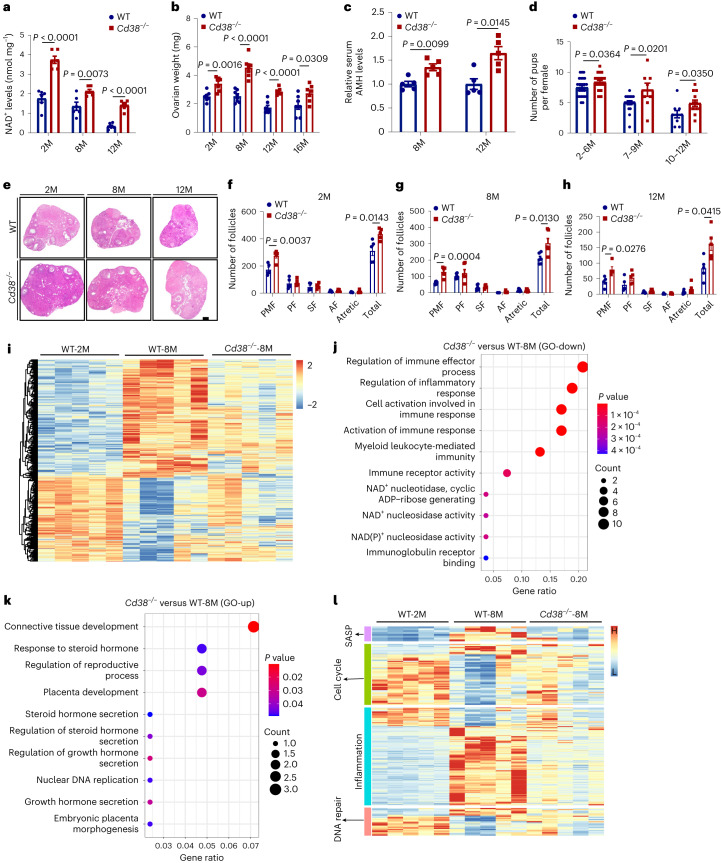
CD38 deletion increased ovarian NAD^+^ levels and improved ovarian follicle reserve, increased reproductive ability of aging mice and shifted the ovarian transcriptome toward a more youthful profile. **a**, Ovarian NAD^+^ levels in WT and *Cd38*^−/−^ mice at different ages (*n* = 6 mice for each group). **b**, Ovarian weight changes during aging in WT and *Cd38*^−/−^ mice (*n* = 7 mice for each group). **c**, Mean serum AMH levels assessed by an ELISA in ovaries from 8-month-old and 12-month-old WT and *Cd38*^−/−^ mice (*n* = 4 or 5 mice for each group). **d**, Mean litter size from WT and *Cd38*^−/−^ mice (*n* = 25 mice for 2–6 months in each group, *n* = 18 for 7–9 months in WT group, *n* = 8 for 7–9 months in *Cd38*^−/−^ group, *n* = 9 for 10–12 months in WT group, *n* = 12 for 10–12 months in *Cd38*^−/−^ group). **e**, Representative hematoxylin-and-eosin-stained ovarian sections from 2-month-old, 8-month-old and 12-month-old WT and *Cd38*^−/−^ mice. Scale bar, 100 μm. **f**–**h**, Numbers of ovarian follicles at different stages, including the primordial follicle (PMF), the primary follicle (PF), the secondary follicle (SF) and the antral follicle (AF), were monitored in WT and *Cd38*^−/−^ mice at different ages (2 months (**f**), 8 months (**g**), 12 months (**h**)) (*n* = 5 or 6 mice for each group). **i**, Heat map of ovarian DEGs between 2-month-old and 8-month-old mice, together with those genes expressed in 8-month-old *Cd38*^−/−^ mice. Genes with log_2_FC > 1 and adjusted *P* value (by the Benjamini–Hochberg method) < 0.05 were considered DEGs (*n* = 5 ovaries for each group). **j**,**k**, GO analysis identified downregulated (**j**) and upregulated (**k**) genes in different pathways between 8-month-old WT and *Cd38*^−/−^ mice. *P* values were calculated by Fisher’s exact test. **l**, Heat map showing the DEGs in the ‘cell cycle’, ‘DNA repair’, ‘inflammation’ and ‘SASP’ pathways in ovaries from 2-month-old WT as well as 8-month-old WT and *Cd38*^−/−^ mice. Data are presented as the mean ± s.e.m. *P* value was determined by the unpaired, two-tailed *t*-test between the two groups. Source data

**Extended Data Fig. 2:**
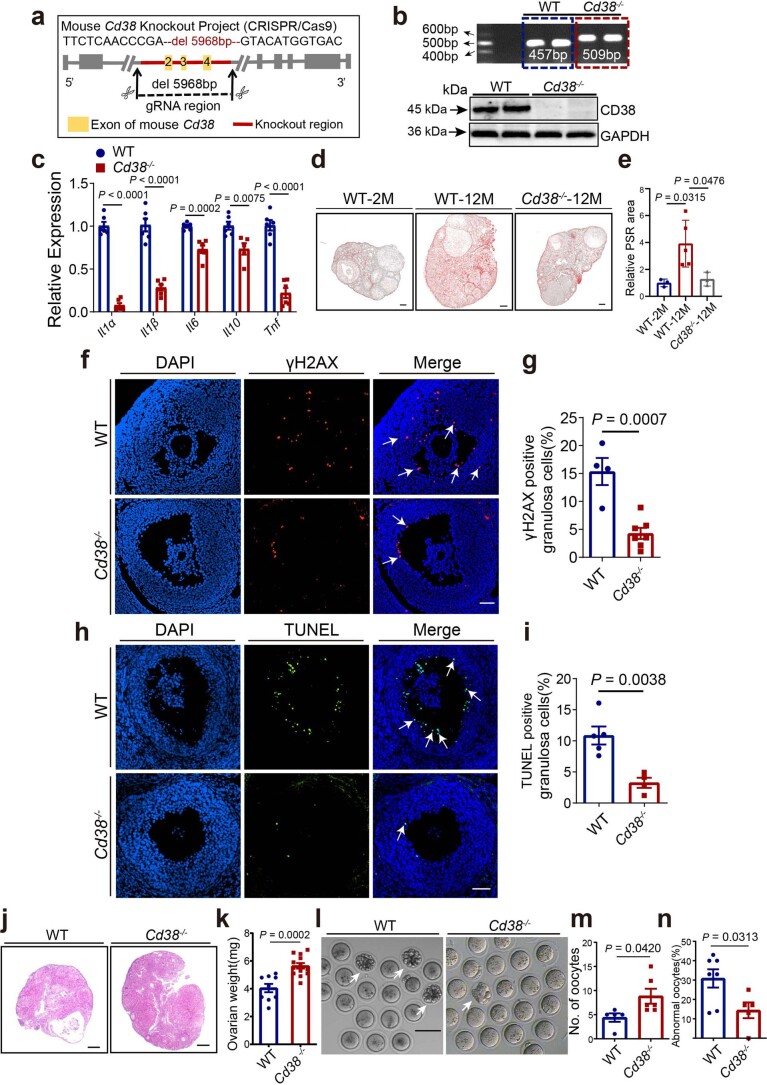
CD38 deletion reduced ovarian aging and improved ovulation potential in aged mice. **a**. Schematic diagram of the generation of *Cd38* knockout mice using CRISPR/Cas9. **b**. PCR genotyping and western blotting confirmed the absence of the CD38 protein in *Cd38*^*−/−*^ ovaries compared with the presence in WT ovaries. **c**. Expression of inflammation-associated factors in ovaries from 8-month-old WT and *Cd38*^*−/−*^ mice determined by RT-PCR (n = 6 for each group). **d**. Representative images of ovarian fibrosis in different from 2-month-old, 12-month-old WT and *Cd38*^*−/−*^ mice by PSR staining of collagen. Bar = 100μm. **e**. PSR positive area was calculated relative to 2-month-old mice. **f**. γH2AX staining of ovarian sections from 12 months old WT and *Cd38*^*−/−*^ mice. Arrows indicated the cells with DNA damage. Bar = 100μm. **g**. Quantitative analysis of γH2AX-positive cells in ovarian sections (n= 4-7 for each group). **h**. TUNEL staining of ovarian sections from 12-month-old WT and *Cd38*^−/−^ mice. Arrows indicated the apoptotic cells. Bar = 100μm. **i**. Quantitative analysis of TUNEL-positive cells in ovarian sections (n = 4-5 for each group). **j**. Representative morphology of ovarian sections from 12-month-old WT and *Cd8*^*−/−*^ mice pretreated with gonadotropins (n = 3-5 mice for each group). Bar = 100μm. **k**. Ovarian weights of WT and *Cd38*^*−/−*^ mice after gonadotropin treatment (n = 9–13 mice per group). **l**. Representative images of oocytes from 12-month-old WT and *Cd38*^*−/−*^ mice. Arrows indicate abnormal oocytes showing fragmentation. Bar = 100μm. **m**. Number of ovulated oocytes from 12-month-old WT and *Cd38*^*−/−*^ mice after gonadotropin-induced ovulation (n = 5–6 mice per group). Bar = 100μm. **n**. Percentage of abnormal oocytes from 12-month-old WT and *Cd38*^*−/−*^ mice (n = 5–7 mice per group). Data are represented as mean ± s.e.m. *P* value was determined by the unpaired *t*-test, two-tailed. Source data

A *Cd38*-deficient mouse model lacking exons 2–4 of the *Cd38* gene was generated using the CRISPR–Cas9 approach to evaluate the impact of CD38 on female fertility (Extended Data Fig. 2a). PCR and western blotting analysis confirmed the *Cd38* mutation and the absence of CD38 protein in the ovaries of *Cd38*^−/−^ mice (Extended Data Fig. 2b). As shown in Fig. 3a, an increase in the ovarian NAD^+^ content was observed in *Cd38*-deficient mice compared with controls at 2, 8 and 12 months of age. Ovarian weight was also higher in *Cd38*^−/−^ versus wild-type (WT) mice (Fig. 3b) at all ages tested. In addition, serum AMH levels were higher in 8-month-old and 12-month-old *Cd38*^−/−^ mice than in their WT littermates (Fig. 3c). Comparison of the reproductive potential between *Cd38*^−/−^ and control mice showed a notably larger litter size from *Cd38*^−/−^ mice compared with controls at young (2–6 months), middle-aged (7–9 months) and aged (10–12 months) mice (Fig. 3d).

Ovarian reserve was analyzed by counting the number of follicles at different developmental stages. Representative images of *Cd38*^−/−^ and WT ovarian sections from mice at different ages are shown in Fig. 3e. The numbers of primordial and total follicles were notably higher in *Cd38*^−/−^ ovaries than in WT ovaries from 2-month-old (Fig. 3f), 8-month-old (Fig. 3g) and 12-month-old (Fig. 3h) mice. However, no differences were found in the number of primary, secondary, antral and atretic follicles between *Cd38*^−/−^ and WT ovaries (Fig. 3f–h). Additionally, CD38 deletion reduced the expression of inflammation-related genes (Extended Data Fig. 2c). Given a prior study indicating fibrosis in the ovarian stromal compartment affecting oocyte release^38^, we examined ovarian fibrosis in aged mice using picrosirius red (PSR) staining. Notably, CD38 deletion led to decreased collagen accumulation in stromal compartment in the aged ovary (Extended Data Fig. 2d,e). CD38 deletion also increased DNA damage (Extended Data Fig. 2f,g) and apoptosis (Extended Data Fig. 2h,i) in follicles in aged (12-month-old) mice compared with WT controls. Following gonadotropin treatment to induce ovulation, 12-month-old *Cd38*^−/−^ mice displayed larger ovaries compared to age-matched WT mice (Extended Data Fig. 2j,k), indicating enhanced ovulatory potential in aged *Cd38*^−/−^ mice. An increased number of ovulated oocytes and a decreased rate of oocytes with fragmentation were observed in 12-month-old *Cd38*^−/−^ mice compared with age-matched control mice (Extended Data Fig. 2l–n). These observations suggested that disruption of CD38 improved the fecundity in female mice.

### CD38 deletion reversed ovarian aging-related gene expression

**Extended Data Fig. 3:**
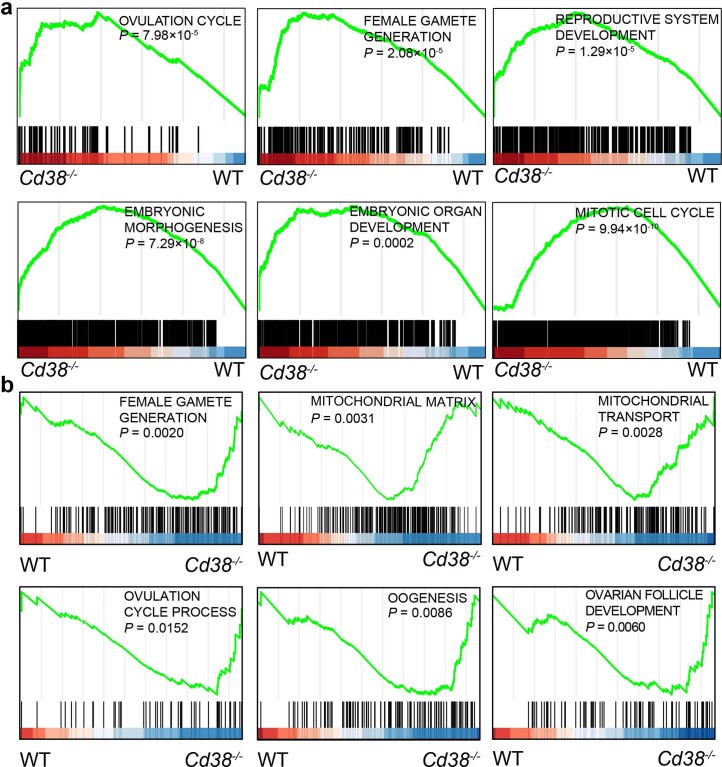
GSEA enrichment analysis for the pathways related to ovarian function and mitochondrial functions. **a**. GSEA analysis showing that the pathways of ovulation cycles, female gamete generation, reproductive system development, embryonic morphogenesis, embryonic organ development, and mitotic cell cycle were enriched in 8-month-old *Cd38*^*−/−*^ compared to age-matched WT mice. **b**. GSEA analysis showed the pathways of female gamete generation, mitochondrial matrix, mitochondrial transport, ovulation cycle process, oogenesis, and ovarian follicle development in 12-month-old *Cd38*^*−/−*^ compared to age-matched WT mice. *P* values were calculated based on Fisher′s Exact Test.

**Extended Data Fig. 4:**
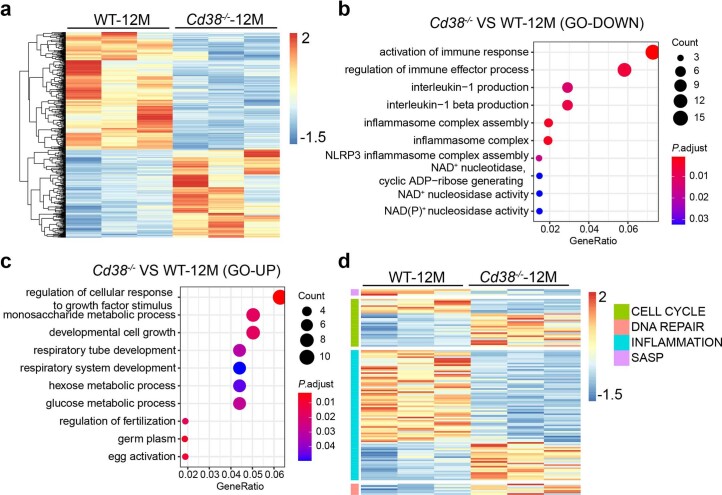
CD38 deficiency shifted the ovarian transcriptome towards a more youthful state in 12-month-old mice. **a**. Heatmap representation of ovarian DEGs between 12-M-old WT and *Cd38*^*−*/−^ mice. **b**. and **c**. GO analysis identified downregulated (b) and upregulated (c) genes in enriched pathways in 12-month-old *Cd38*^*−/−*^ mice. *P* values were calculated by Fisher′s Exact Test and adjusted by the Benjamini-Hochberg method. **d**. Heatmap showing the DEGs in cell cycle, DNA repair, inflammation, and SASP pathways in ovaries from 12-month-old WT and *Cd38*^*−*/−^ mice.

We performed transcriptome analysis of ovaries from *Cd38*^−/−^ and WT mice in the diestrus stage at different ages using bulk RNA-seq to assess whether a longer reproductive lifespan in *Cd38*-null mice was accompanied by molecular changes. The heat map data showed that the transcriptome profile of ovaries from middle-aged (8-month-old) WT mice was different from that of ovaries from young (2-month-old) WT mice (Fig. 3i and Supplementary Table 1). Of interest, the transcriptome profile of ovaries from middle-aged *Cd38*^−/−^ mice clustered in an intermediate position between the middle-aged and young WT ovaries (Fig. 3i and Supplementary Table 3). The expression of key upregulated and downregulated genes in ovaries from each group was further confirmed using real-time RT‒PCR (Supplementary Fig. 2). GO analysis revealed that downregulated genes in middle-aged *Cd38*^−/−^ ovaries were mostly enriched in pathways related to inflammation and NAD^+^ metabolism (Fig. 3j). Upregulated genes in the middle-aged *Cd38*^−/−^ ovaries were enriched in the processes of reproductive development (Fig. 3k). Analysis of DEGs in senescence-related pathways, including ‘SASP’, ‘cell cycle’, ‘DNA repair’ and ‘inflammation’, showed that the transcriptional signature of 8-month-old *Cd38*^−/−^ ovaries for these gene sets was intermediate between 2-month-old and 8-month-old WT ovaries (Fig. 3l). Gene set enrichment analysis (GSEA) further demonstrated that deletion of CD38 improved ovarian function, as shown by enrichment of pathways such as ‘female gamete generation’ and ‘reproductive system development’ (Extended Data Fig. 3a). Ovarian transcriptome analysis was also performed with 12-month-old *Cd38*^−/−^ and age-matched WT mice. Similarly, the results showed that deletion of CD38 partially reversed the age-related changes in pathways related to ovarian function, mitochondria, inflammation and SASP (Extended Data Figs. 3b and 4a–d and Supplementary Table 4). These results suggest that CD38 deletion prevents ovarian senescence.

**Fig. 4:**
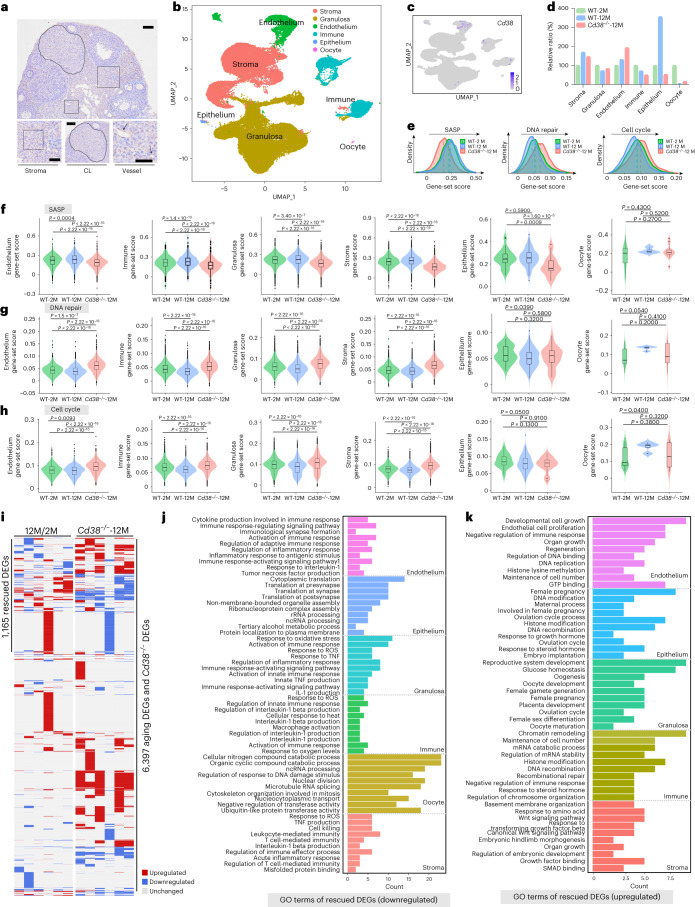
CD38 deletion reversed aging-related cell-type-specific gene expression changes. **a**, IHC of CD38 in ovarian sections from 2-month-old WT mice. Bottom: magnification of images showing CD38 localizes in the stroma, corpora lutea (CL) and vessel. Scale bars, 50 μm (top) and 25 μm (bottom). **b**, UMAP plot showing the six ovarian cell types. **c**, UMAP plots showing the *Cd38* expression in the six ovarian cell types. **d**, Proportions of the six major cell types in the ovaries from 2-month-old and 12-month-old WT and 12-month-old *Cd38*^−/−^ mice by scRNA-seq. **e**, Ridge plot showing the shift of SASP, DNA repair and cell cycle gene-set scores with age and CD38 deletion. **f**–**h**, Violin plots showing the SASP (**f**), DNA repair (**g**) and cell cycle (**h**) set scores in each cell type from different groups. Statistical analysis was performed using two-sided Wilcoxon rank-sum tests. **i**, Heat maps illustrating the distribution of DEGs in each specific cell type present in the ovaries from 2-month-old, 12-month-old WT and 12-month-old *Cd38*^−/−^ mice. Aging DEGs refer to genes that exhibited changes between the 12-month-old and 2-month-old groups, specifically in the context of aging. *Cd38*^−/−^ DEGs indicate genes that displayed alterations in the 12-month-old *Cd38*^−/−^ group compared with the age-matched WT group. Rescued DEGs are genes that demonstrated inverse changes in the *Cd38*^−/−^ DEG group as compared with the aging DEG group. **j**, Representative GO terms and pathways enriched in CD38 deletion rescued DEGs that are downregulated, identified through functional enrichment analysis. **k**, Representative GO terms and pathways enriched in CD38 deletion rescued DEGs that are upregulated, identified through functional enrichment analysis.

In situ immunohistochemistry (IHC) staining of CD38 on ovarian sections revealed predominant localization of CD38 in the stroma, corpora lutea and vessel (Fig. 4a). These findings are consistent with those reported in a recent study, which highlighted the role of CD38 in regulating ovarian function and fecundity through NAD^+^ metabolism^39^. To gain further insights into the impact of aging and CD38 deletion on cell-type transcriptional profiles, we generated single-cell atlases for young (2-month-old) WT mice, old (12-month-old) WT mice and *Cd38*^−/−^ (12-month-old) mice. After stringent cell filtration, 64,479 cells were retained for subsequent analyses. Global ovarian cell populations were visualized using uniform manifold approximation and projection (UMAP), which led to the identification of six distinct cell types based on specific marker expression (Supplementary Table 5): granulosa cells, stromal cells, immune cells, endothelial cells, epithelial cells and oocytes (Fig. 4b). Analysis of *Cd38* expression levels across the six ovarian cell types revealed prominent expression in endothelial cells and immune cells (Fig. 4c). The proportions of cell types captured by single-cell sequencing were comparable across young, old and CD38-deleted old mice. As shown in Fig. 4d, most of the cell types exhibited changes in proportions during aging, which were reversed after deletion of CD38, including granulosa cells, stromal cells, epithelial cells and oocytes. Consistent with the findings from bulk-seq data of the entire ovary, scRNA-seq data also showed upregulation of SASP-related genes and downregulation of DNA repair and cell cycle-related genes in the aging ovary (Fig. 4e). Specifically, in the aged ovary after CD38 deletion, the expression of SASP-related genes was repressed in endothelial, immune, granulosa, stromal and epithelial cells. However, all these changes were reversed upon CD38 deletion (Fig. 4f). Conversely, DNA repair and cell cycle-related genes exhibited higher expression in endothelial, immune, granulosa and stromal cells in the aged CD38-deleted ovary compared to WT controls (Fig. 4g,h). To elucidate the molecular events associated with aging and CD38 deletion, we identified DEGs between old and young ovaries and between *Cd38*^−/−^ and old ovaries, referred to as ‘aging DEGs’ and ‘*Cd38*^−/−^ DEGs,’ respectively. CD38 deletion rescued 1,165 genes and the upregulated and downregulated DEGs in each cell type are shown in Fig. 4i. GO term analysis revealed that CD38 deletion reduced the expression of aging-induced genes related to immune response pathways in endothelial, granulosa, immune and stromal cells (Fig. 4j). As expected, CD38 deletion increased the expression of aging-induced downregulated genes related to cell growth and cell proliferation in endothelial cells, female pregnancy in epithelial cells, ovulation cycle and oocyte maturation in granulosa cells, recombinational repair in immune cells and regulation of embryonic development pathways in stromal cells (Fig. 4k). These results suggest the presence of a coordinated intercellular regulation of ovarian function and the deletion of CD38 improved overall ovarian functions.

### Restoration of transcriptional regulatory networks and aging-related aberrant cell–cell communication patterns in the ovary after CD38 deletion

**Fig. 5:**
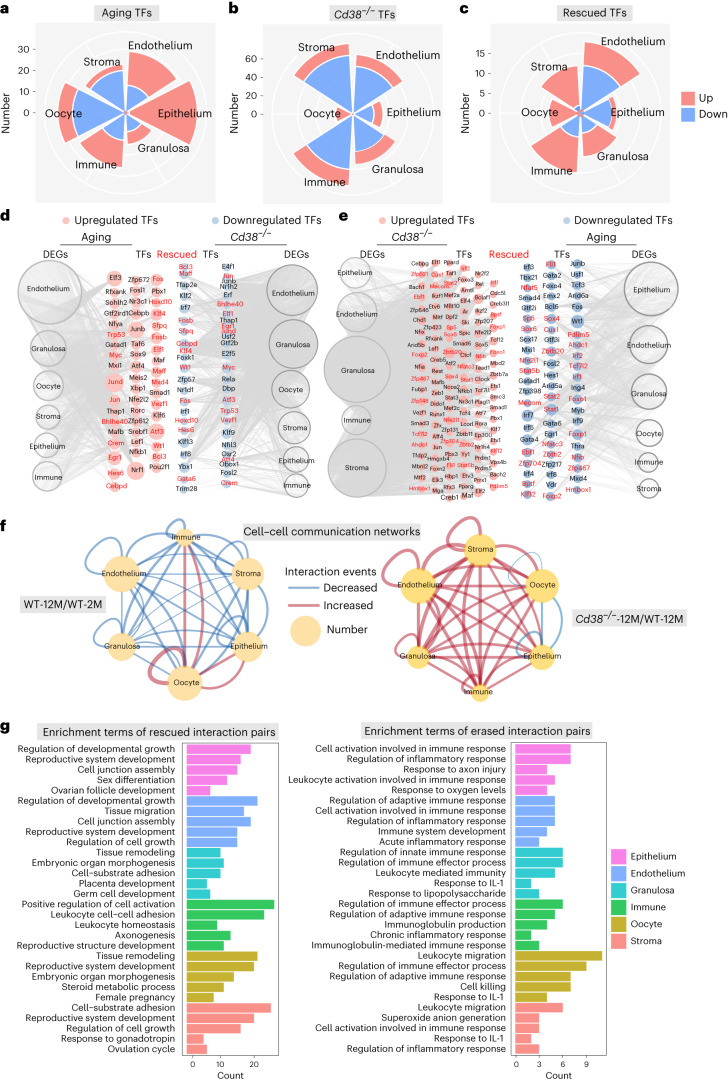
CD38 deletion rescued aging-related changes in core regulatory TFs and ligand–receptor interactions between different cell types. **a**–**c**, Rose diagrams showing the numbers of changes of TFs with aging (**a**, aging TFs), CD38 deletion (**b**, *Cd38*^−/−^ TFs) and rescued TFs after CD38 deletion (**c**, rescued TFs) in the six cell types. **d**,**e**, Visualization of CD38 deletion downregulated (**d**) and upregulated (**e**) rescued TFs in the network. The internal nodes represent TFs, with gray circular edges indicating downstream target DEGs influenced by these TFs. Node sizes are proportional to the number of associated DEGs. The rescued TFs are shown in red. **f**, Network diagrams illustrate alterations in ligand–receptor interaction events across distinct cell types in the ovaries of 2-month-old and 12-month-old WT mice (left), as well as in 12-month-old *Cd38*^−/−^ and WT mice (right). Cell–cell communication is represented by interconnected lines, with line thickness reflecting a positive correlation with the quantity of ligand–receptor interaction events. **g**, GO enrichment of rescued (left) and erased (right) interaction pairs in each cell type.

To understand the effect of CD38 on the transcriptional regulatory networks underlying ovarian aging, we used SCENIC to predict the core TFs responsible for regulating ovarian aging after CD38 deletion. The changes of core TFs with aging and CD38 deletion are shown in Fig. 5a,b. The CD38 deletion-rescued TF DEGs are shown in Fig. 5c. The results revealed that the rescued TFs and their target DEGs were found in all six types of cells. Specifically, Myc, a TF that regulates the expression of many genes involved in critical cellular functions and has been linked to longevity^40^, showed increased expression during aging but was downregulated after CD38 deletion (Fig. 5d). Furthermore, CEBPD, a core transcriptional factor associated with chronic inflammatory diseases^41^, also exhibited increased expression during aging and decreased expression after CD38 deletion (Fig. 5d). Additionally, Foxo1, a TF that plays essential roles in cellular homeostasis^42^, was downregulated during aging but upregulated in the *Cd38*-knockout ovary (Fig. 5e). These findings suggest that CD38 deletion reshapes the aging-associated transcriptional regulatory networks in the ovary, impacting genes and pathways involved in inflammation processes and ovarian functions.

To investigate the impact of CD38 deletion on cell–cell communications in the ovary during aging, we initially analyzed the ligand–receptor interactions among the ovarian cell types. The results showed a general decline in cell–cell communications in most cell types in the aged ovary (Fig. 5f). However, interestingly, in the aged CD38 deletion ovary, several types of cell–cell communications between specific cell types were actually enhanced (Fig. 5f). To gain further insights, we conducted GO enrichment analyses on the interaction pairs for each cell type. The data showed that CD38 deletion rescued important interactions in various cellular processes (Fig. 5g). For instance, in epithelial cells, endothelial cells, stromal cells and oocytes, CD38 deletion positively enhanced cell–cell communications involved in reproductive system development. In granulosa cells, CD38 deletion was associated with improved interactions related to germ cell development. Additionally, in immune cells, CD38 deletion enhanced leukocyte cell–cell adhesion. Altered intercellular communication is an integrative hallmark of aging^43^. These results demonstrated that CD38 deletion notably reversed the disrupted cell–cell communication patterns associated with ovarian aging.

### CD38 deletion ameliorated age-related oocyte quality decline

**Fig. 6:**
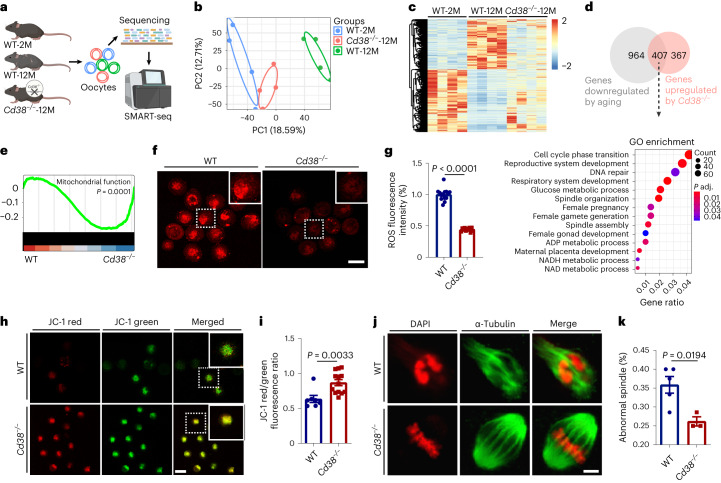
Single-cell sequencing showed that the oocyte transcriptome of 12-month-old *Cd38*^−/−^ mice resembled that of WT mice at 2 months of age, and age-related oocyte quality decline was attenuated after the deletion of CD38. **a**, Workflow of single-oocyte sequencing by SMART-seq. Oocytes were retrieved from young (2-month-old) and aged (12-month-old) mice together with those from 12-month-old *Cd38*^−/−^ mice. **b**, PCA of single-oocyte RNA transcriptomes from young (2-month-old) and aged (12-month-old) WT and *Cd38*^−/−^ mice (*n* = 4 oocytes for each group). **c**, Heat map showing gene expression of oocytes from young and aged WT and *Cd38*^−/−^ mice. **d**, Venn diagram showing the overlap between age-related downregulated genes (aged versus young) and upregulated genes after CD38 deletion. GO analysis showed 407 DEGs in enriched pathways. *P* values were calculated by Fisher’s exact test. **e**, GSEA showing the enrichment of mitochondrial functions in oocytes from 12-month-old *Cd38*^−/−^ oocytes. *P* values were calculated based on Fisher’s exact test. **f**, Representative images of mitochondrial ROS levels in oocytes detected by MitoSOX staining in 12-month-old WT and *Cd38*^−/−^ oocytes. Scale bar, 100 μm. **g**, The fluorescence intensity of ROS signals was measured in oocytes from 12-month-old WT and *Cd38*^−/−^ mice (*n* = 11–14 mice for each group). **h**, Representative images of JC-1-stained MII oocytes from 12-month-old WT and *Cd38*^−/−^ mice. **i**, The mitochondrial membrane potential was calculated as the ratio of JC-1 red to JC-1 green signals in oocytes from 12-month-old WT and *Cd38*^−/−^ mice (*n* = 7–13 oocytes for each group). **j**, Representative images of spindle assembly and chromosome alignment in oocytes from 12-month-old WT and *Cd38*^−/−^ mice. **k**, Mean abnormal spindle ratios in oocytes from 12-month-old WT and *Cd38*^−/−^ mice (*n* = 3–5 mice for each group). Scale bar, 5 μm. Data are presented as the mean ± s.e.m. *P* value was determined by the unpaired two-tailed *t*-test between the two groups. Source data

**Extended Data Fig. 5:**
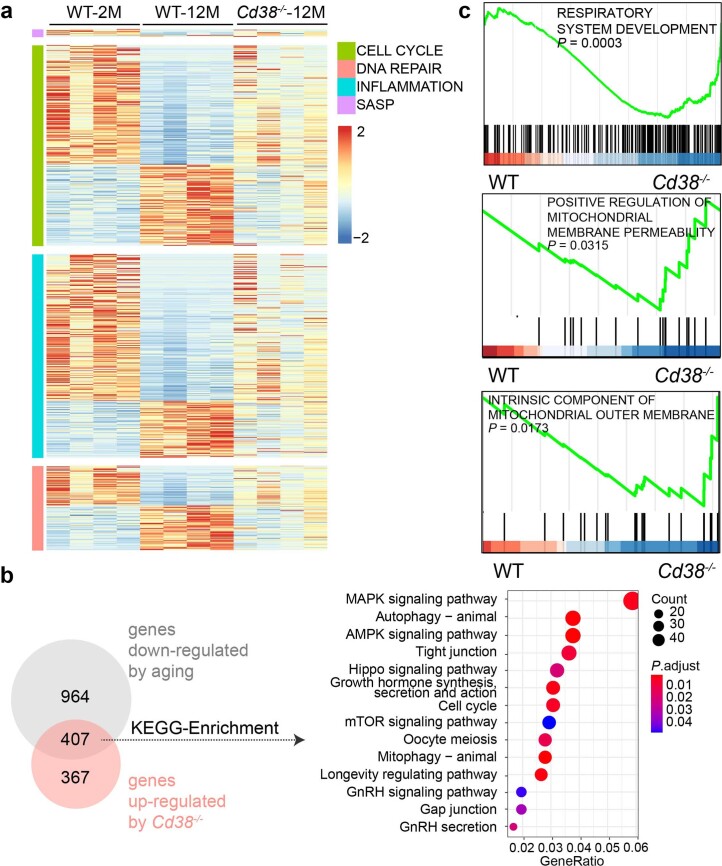
Single-oocyte sequencing revealed that CD38 deletion down-regulates senescence-related pathways and enhanced mitochondrial functions. **a**. Heatmap showing the DEGs enriched in aging-related pathways of senescence-associated secretory phenotype (SASP), cell cycle, inflammation, and DNA repair in young, aged WT and aged *Cd38*^*−/−*^ oocytes. **b**. Venn diagram showing the overlap between age-related downregulated genes (Aged vs. Young) and upregulated after deletion of CD38. 407 DEGs were found, and KEGG analyses showed related pathways of the 407 DEGs. *P* values were calculated by Fisher′s Exact Test and adjusted by the Benjamini-Hochberg method. **c**. GSEA enrichment plots of GO pathways related to mitochondrial functions in 12-month-old WT and *Cd38*^*−/−*^ oocytes. *P* values were calculated based on Fisher′s Exact Test.

Metaphase II (MII) oocyte quality decline is a hallmark of ovarian senescence^44,45^. Due to the larger size of MII oocytes, scRNA-seq for the whole-ovary methods was unable to provide transcriptomic data for these oocytes. To investigate the potential impact of CD38 deletion on the quality of aging oocytes, we conducted single-cell transcript analysis on ovulated MII oocytes from young (2-month-old) and old (12-month-old) *Cd38*^−/−^ and WT mice using the SMART-seq method (Fig. 6a). Principal component analysis (PCA) was performed, and PC1 revealed distinct expression patterns between young and old oocytes (Fig. 6b and Supplementary Table 6). Notably, the oocyte gene transcription profile of 12-month-old *Cd38*^−/−^ mice was found to be intermediate between that of 2-month-old and 12-month-old WT oocytes. Furthermore, the heat map indicated that the DEGs following oocyte aging were partially reversed in 12-month-old *Cd38*^−/−^ mice (Fig. 6c), particularly concerning senescence-related pathways (Extended Data Fig. 5a). Among the identified DEGs, 407 genes were found to be downregulated during aging but upregulated following CD38 deletion (Fig. 6d and Supplementary Table 7). GO and GSEA analyses revealed that these DEGs were associated with processes such as ‘DNA repair’, ‘reproductive system development’, ‘cell cycle phase transition’, ‘spindle organization’, ‘female gamete generation’, ‘oocyte meiosis’ and ‘mitochondrial membrane permeability’ (Fig. 6d and Extended Data Fig. 5b,c). Given the close relationship between mitochondrial dysfunction and declining oocyte quality with aging, these findings suggest a potential role of CD38 in modulating oocyte aging and mitochondrial function^46,47^. Considering that mitochondrial dysfunction is closely related to declining oocyte quality with age, mitochondria-related gene expression (Supplementary Table 8) was further analyzed by GSEA, which revealed that these genes were upregulated in *Cd38*^−/−^ oocytes compared with age-matched WT oocytes (Fig. 6e and Extended Data Fig. 5c).

The ROS contents in oocytes were assessed to investigate the effects of CD38 deletion on oocyte quality during aging. Deletion of CD38 reduced ROS accumulation in aging oocytes (Fig. 6f,g). As shown in Fig. 6h,i, the mitochondrial membrane potential (ΔΨm) in oocytes was notably increased in 12-month-old *Cd38*^−/−^ mice compared with WT mice, indicative of improvement in mitochondrial activity. We further investigated whether age-related meiotic defects in oocytes could be improved with the loss of CD38. As shown in Fig. 6j,k, the abnormal spindle/chromosome structural ratios in WT oocytes of aged mice were decreased in *Cd38*^−/−^ counterparts, indicating that loss of CD38 reduced meiotic defects. These results revealed that CD38 deficiency ameliorated the age-related deterioration of oocyte quality.

### CD38 deletion prevented inflammation-induced follicle loss

**Fig. 7:**
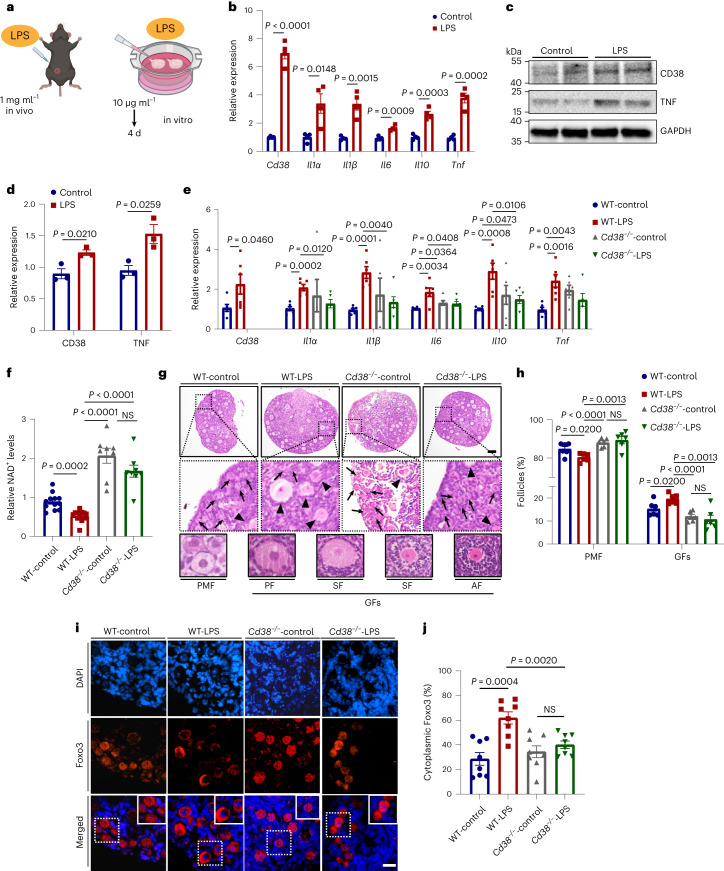
Inflammation-induced primordial follicle loss was mediated by activation of CD38 and a decline in NAD^+^ levels. **a**, Workflow showing LPS treatment in vivo and in vitro. **b**, Expression levels of *Cd38* and inflammation-associated genes (*Il1a*, *Il1b*, *Il6*, *Il10* and *Tnf*) by real-time RT‒PCR for ovaries 24 h after injection of LPS (*n* = 4 for each group). **c**, Western blotting analysis of CD38 and TNF protein levels in ovaries 24 h after LPS injection. **d**, Relative expression of each protein relative to GAPDH levels (*n* = 3 for each group). **e**, Expression of *Cd38* and inflammation-associated factors in cultured ovaries from 10-day-old WT-control, LPS-treated WT, *Cd38*^−/−^-control and LPS-treated *Cd38*^−/−^ mice (*n* = 6 for each group). **f**, NAD^+^ levels in cultured WT-control, LPS-treated WT, *Cd38*^−/−^-control and LPS-treated *Cd38*^−/−^ ovaries (*n* = 8 experimental replicates, each using 2 or 3 ovaries). **g**, Representative images of ovarian sections from cultured WT-control, LPS-treated WT, *Cd38*^−/−^-control and LPS-treated *Cd38*^−/−^ ovaries. The enlarged pictures show primordial follicles (arrows) and growing follicles (GFs; arrowheads) in ovaries from the different groups. The bottom images are representative of primordial follicles and GFs (primary, secondary and antral follicles) in WT mice (*n* = 6 ovaries for each group). Scale bar, 100 μm. **h**, Percentages of primordial follicles and growing follicles in ovaries for each group. **i**, Immunofluorescence staining of Foxo3 in ovarian sections cultured for 4 d for the different groups. Scale bar, 20 μm. **j**, Percentages of nuclear export of Foxo3 in primordial follicles from different groups (*n* = 8 ovaries for each group). Data are presented as the mean ± s.e.m. *P* value was determined by unpaired two-tailed *t*-test between the two groups. NS, not significant. Source data

The above findings indicated that inflammation-related pathways were activated and that CD38 expression was increased in middle-aged ovaries. To focus on the relationship between inflammation activation and CD38 expression, 8-month-old mice were treated with 1 mg ml^−1^ lipopolysaccharide (LPS) in vivo (Fig. 7a). After 24 h, LPS-treated mice had increased expression of inflammatory cytokines in the ovary (Fig. 7b–d). In vitro, ovaries from 10-day-old mice were cultured with LPS (10 μg ml^−1^) for 4 d, at which point they exhibited elevated expression of inflammation-associated genes (Fig. 7e). Additionally, LPS treatment increased ovarian CD38 expression in vivo and in vitro (Fig. 7b–e).

We further investigated the effect of CD38 and LPS on ovarian NAD^+^ levels. Ovaries from 10-day-old *Cd38*^−/−^ mice cultured and treated with LPS showed higher expression of inflammation-related genes, but ovarian NAD^+^ levels were not affected, in contrast to LPS-treated WT ovaries, which showed a reduction in NAD^+^ levels (Fig. 7f). Histological analysis and follicle counting were performed to investigate the effects of inflammation-induced CD38 activation on the ovarian primordial follicle reserve pool (Fig. 7g). As shown in Fig. 7h, LPS-treated ovaries had a notably reduced proportion of primordial follicles compared with that of controls, whereas the proportion of growing follicles (primary, secondary and antral follicles) was larger in the LPS-treated ovaries. However, these changes were not observed in the ovaries after deletion of CD38 (Fig. 7h).

The TF Foxo3 is required for suppression of primordial follicle activation, and nuclear exclusion of Foxo3 in oocytes results in primordial follicle activation^48,49^. The intracellular location of Foxo3 in primordial follicle oocytes of cultured ovary sections was analyzed in the different groups. As shown in Fig. 7i, LPS treatment increased the nuclear export of Foxo3 in primordial follicle oocytes. Quantitative analyses indicated that 61.87% of primordial follicle oocytes exhibited Foxo3 export (Fig. 7j) in WT LPS-treated ovaries. In contrast, only 40.14% of primordial follicle oocytes showed Foxo3 export in *Cd38*^−/−^ LPS-treated ovaries. These results suggested that the inflammation-induced decrease in the primordial follicle pool was mediated by CD38.

### 78c treatment rescued age-related female fertility decline

**Fig. 8:**
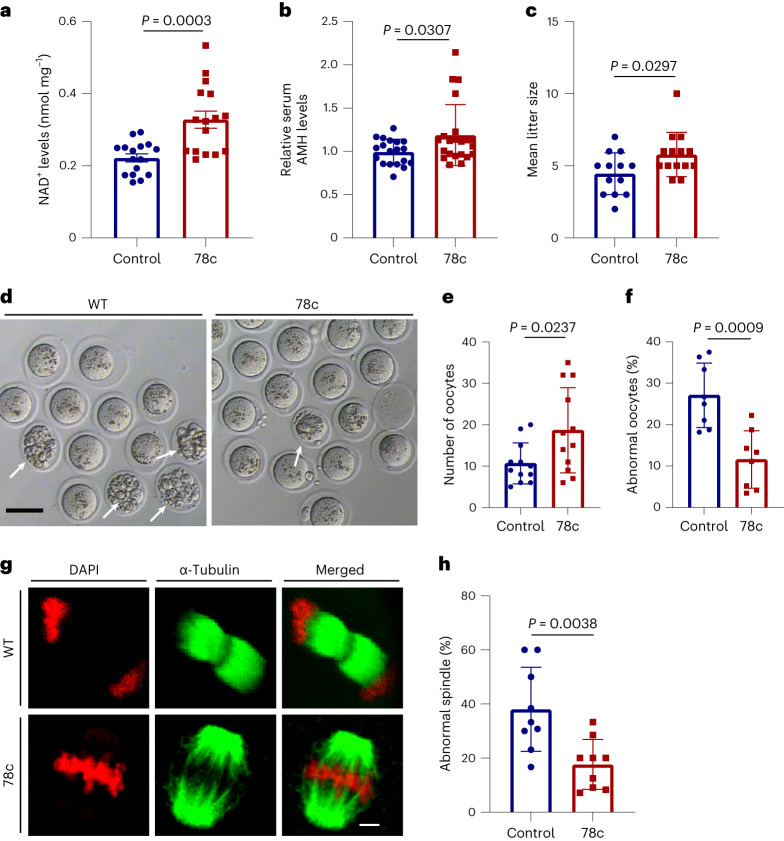
Pharmacological inhibition of CD38 improved female fertility in middle-aged mice. **a**, Ovarian NAD^+^ levels in middle-aged mice with or without 78c treatment for 8 d (*n* = 16 mice for each group). **b**, Serum AMH levels in control and 78c-treated middle-aged mice for 3 weeks (*n* = 19–22 mice for each group). **c**, Mean litter size of middle-aged mice with or without 78c treatment (*n* = 13 or 14 mice for each group). **d**, Representative images of MII oocytes from superovulated control and 78c-treated middle-aged mice (*n* = 12 mice for each group). Arrows indicate oocytes with fragmentation. Scale bar, 100 μm. **e**, Mean ovulated oocyte numbers collected from control and 78c-treated middle-aged mice. **f**, The proportion of abnormal oocytes in each group (*n* = 8 mice for each group). **g**, Representative images of the spindle morphology and chromosome alignment in MII oocytes from control and 78c-treated middle-aged mice. Scale bar, 5 μm. **h**, The proportion of abnormal spindles in oocytes from each group (*n* = 9 mice for each group). Data are presented as the mean ± s.e.m. *P* value was determined by unpaired two-tailed *t*-test between the two groups. Source data

**Extended Data Fig. 6:**
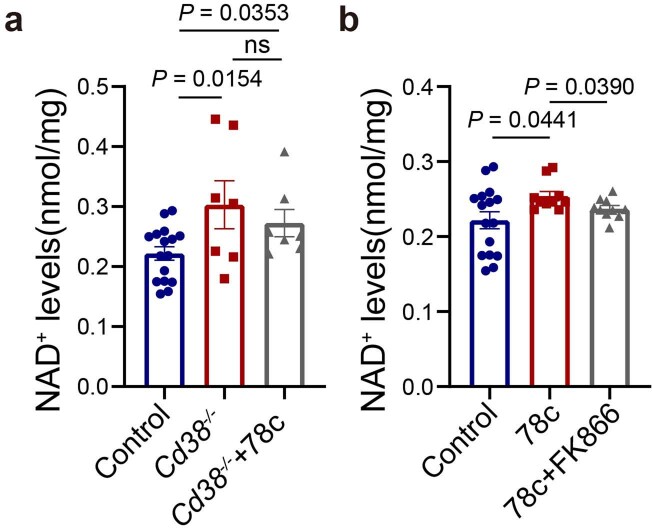
NAD^+^ levels in the ovaries from different groups. **a**. Ovarian NAD^+^ levels in the control (WT) and *Cd38*^*−/−*^ mice treated with 78c for 8 days. (n = 16 for control, n = 7 for *Cd38*^*−/−*^ and *Cd38*^*−/−*^+78c group). **b**. Ovarian NAD^+^ levels in the WT, 78c-treated and 78c+FK866-treated mice for 3 days. (n = 16 for control, n = 10 for 78c and 78c+FK866 group). Data are represented as mean ± s.e.m. *P* value was determined by the unpaired *t*-test, two-tailed. Source data

We conducted further investigations to assess the impact of a CD38 (ADP ribosyl cyclase/hydrolase) inhibitor, 78c, on the ovarian function of middle-aged female mice. Initially, we administered 78c injections to 8-month-old mice for 8 d to verify its effect on ovarian NAD^+^ levels. We observed a significant increase in ovarian NAD^+^ levels in middle-aged mice following treatment with 78c (Fig. 8a). However, this effect was not observed in *Cd38*-knockout mice (Extended Data Fig. 6a), highlighting the essential role of CD38 in mediating 78c’s actions on ovarian NAD^+^ levels. Moreover, we also found that administration of FK866, an inhibitor of nicotinamide phosphoribosyltransferase (NAMPT) for 3 d, could reduce the 78c-induced increase in ovarian NAD^+^ (Extended Data Fig. 6b). These results demonstrated that the effect of 78c on ovarian NAD^+^ levels was indeed dependent on CD38. To investigate its potential benefits on fertility, we treated 8-month-old mice with 78c for 3 weeks. The 78c-treated mice exhibited elevated serum levels of AMH, a marker for ovarian reserve (Fig. 8b). Furthermore, after mating, the 78c-treated mice showed a notable increase in the mean number of pups compared to the control group (Fig. 8c). Moreover, the 78c-treated mice had approximately twofold more oocytes retrieved after undergoing superovulation induced by gonadotropins, and a reduced proportion of abnormal oocytes showing vacuolization and fragmentation (Fig. 8d–f). Furthermore, the analysis of spindle morphology revealed that 38.03% of MII oocytes from the control group exhibited abnormal spindle/chromosome structures, whereas the 78c-treated group showed only 17.66% with abnormalities (Fig. 8g,h). These findings clearly indicate that short-term treatment with 78c notably improves the fertility of middle-aged female mice, similar to the effect observed with CD38 deletion.

## Discussion

Delayed childbearing is prevalent worldwide, and ovarian senescence occurs earlier than most of the other organs in females. Ovarian function decreased dramatically in middle age, as shown by a decrease in oocyte quality and ovarian reserve^50,51^. We hypothesized that middle-aged mice may be useful for investigating the molecular mechanisms underlying ovarian senescence. Our study showed the transcriptome changes that occur in the ovaries of middle-aged mice when many other organs showed no aging-related gene changes. In particular, gene transcripts in aging-related pathways, including SASP, cell cycle, inflammation and DNA repair, were misregulated in the ovary but not in multiple other organs when comparing middle-aged with young mice. Indeed, increased expression of aging markers, namely, p16 and p21, and inflammation-related factors was observed in the ovary but not in other organs from middle-aged mice. Our findings are consistent with a report classifying the aging-associated alterations in gene expression patterns of different tissues into four stages with ovarian aging occurring in 6-month-old to 12-month-old mice^52^, which is earlier than for most of the other organs.

Importantly, the current study showed that the expression of inflammation-related genes rapidly increased in the middle-aged ovary, accompanied by activation of the NAD^+^ metabolizing enzyme CD38, whereas other key enzymes for NAD^+^ generation and metabolism were not changed in the ovaries from middle-aged mice. The activation of CD38 and inflammation-related transcripts was not observed in other organs. A previous study showed that CD38 levels increased in the liver, adipose tissue, spleen and skeletal muscle in aged (approximately 18-month-old) mice^32^, indicating that the increases in CD38 expression during middle age are likely a key event during ovarian senescence. We and several groups have reported that ovarian NAD^+^ levels decline during aging, whereas boosting NAD^+^ by supplementation with NAD^+^ precursors, such as nicotinamide riboside or nicotinamide mononucleotide, increased ovarian NAD^+^ levels and delayed ovarian aging by improving mitochondrial function^20–23^. The present work found that deletion of CD38 prevented ovarian NAD^+^ decline, extended ovarian lifespan and resulted in increased litter sizes in aged mice. Importantly, increased ovarian follicle reserve was found in aged *Cd38*^−/−^ mice compared with WT mice. Consistent with these findings, higher levels of serum AMH and decreased cell DNA damage and apoptosis were observed in the ovarian follicles of *Cd38*^−/−^ mice than in those of age-matched WT mice. Additionally, aged (12-month-old) *Cd38*^−/−^ mice exhibited a markedly increased response to gonadotropins, which yielded a higher number of healthy preovulatory follicles than those of age-matched WT mice.

To further investigate the impact of CD38 deletion on ovarian function, we conducted bulk RNA-seq and scRNA-seq analyses, revealing notable changes in the transcriptome of the aged ovary, particularly affecting genes related to the senescence pathway, which were largely suppressed in *Cd38*-deficient mice. These results strongly suggest that the earlier onset of ovarian senescence is likely attributed to increased expression of CD38, resulting in a decline in NAD^+^ levels. Additionally, the scRNA-seq data highlighted that CD38 expression was primarily observed in endothelial cells and immune cells. The ovarian stroma encompasses essential components, including immune cells, blood vessels, nerves and the extracellular matrix^53^. In situ immunostaining identified that CD38 protein mainly localized in ovarian stroma. The ovarian stroma not only offers structural support for follicle development but also engages in intricate bidirectional paracrine signaling with the follicles^54^. We observed notable alterations in the transcriptome of various cell types within the ovary upon CD38 deletion, with many genes related to ovarian function pathways being upregulated. Moreover, CD38 deletion facilitated the restoration of core TF expression, consequently rejuvenating the aging-related disruptions in transcriptional regulatory networks. Importantly, we found that CD38 deletion restored the expression of core TFs, leading to the restoration of transcriptional regulatory networks during aging. The ovary is a complex organ composed of multiple cell types that require intricate systemic regulation for follicle development^25^, and alteration of cell–cell communication is considered a hallmark of tissue aging^43^. Our research provides insights into the beneficial effects of CD38 deletion, highlighting its role in restoring core TF expression and cell–cell communication patterns to counteract ovarian aging.

The present study also revealed that CD38 deletion improved oocyte quality, with single-oocyte transcriptomic analysis indicating enhancement of pathways, including ‘DNA repair’ and ‘female gamete generation’, as well as ‘mitochondrial membrane permeability’, in oocytes from *Cd38*^−/−^ mice. Analysis of ROS levels, spindle/chromosome organization and mitochondrial membrane potential demonstrated that CD38 deletion reduced oocyte abnormalities in aged animals. Human female reproductive ability decreases during the fourth decade of life because of age-related changes in oocyte quality and quantity^55^. The accumulation of ROS and mitochondrial dysfunction, together with spindle/chromosome organization failure, are hallmarks of the age-related decline in oocyte quality, which is a major cause of pregnancy loss and developmental disorders in offspring^56^. While our studies primarily focused on the NADase activity of CD38, it is important to acknowledge that CD38 may play roles in various other cellular functions, including cell adhesion, signal transduction and calcium signaling^57^. Notably, despite the absence of observed CD38 expression in oocytes, our research and findings from other research groups consistently demonstrate reduced NAD^+^ levels in aging mouse oocytes^20,22^. Furthermore, senescent cells could induce the upregulation of CD38 mRNA and protein expression, leading to increased CD38-NADase activity in non-senescent cells^58^. These cumulative results further strengthen the proposition that CD38 exerts regulatory control over ovarian aging, operating through both direct mechanisms and indirect influence on cell–cell communication within the ovarian microenvironment.

Most CD38 inhibitors are antibodies, such as Darzalex (daratumumab), which has been approved by the US Food and Drug Administration to treat multiple myeloma patients^59^. However, these antibodies are unable to inhibit intracellular CD38. Recently, the small molecule 78c was found to ameliorate age-related metabolic dysfunction by reversing tissue NAD^+^ decline^60^, thereby increasing the lifespan in mice^61^. The current study further investigated the effects of 78c on ovarian function in mice and showed that 78c treatment increased ovarian NAD^+^ levels. Administration of 78c for 3 weeks also increased serum AMH levels and ovulated oocyte numbers after gonadotropin treatment. In addition, oocyte quality was improved in 78c-treated mice, as evidenced by the reduction in abnormal oocyte morphology and spindle assembly, along with increased litter size. These findings suggest that short-term inhibition of CD38 might be a promising approach for treating infertility in middle-aged cisgender women and other people with ovaries.

As we observed a notable increase in the expression of CD38 and inflammation-related genes in 8-month-old mice, we aimed to elucidate the regulatory relationship between these two factors and explore potential mechanisms contributing to ovarian aging. To investigate this, we induced inflammation using LPS treatment both in vivo and in vitro, which resulted in an increase in ovarian CD38 expression and a reduction in ovarian NAD^+^ levels. These findings were consistent with other reports that indicate inflammation can upregulate CD38 and decrease NAD^+^ levels in other tissues^62,63^. We further demonstrated that inflammation induced a decrease in the primordial follicle pool, consistent with a previous study in rats^64^. In contrast, deletion of CD38 prevented the inflammation-induced loss of the primordial follicle pool, highlighting the crucial role of CD38 in the inflammation-induced decline in ovarian reserve with age.

We also identified substantial shifts in the proportions of most cell types during ovarian aging, and these changes were notably reversed upon CD38 deletion. However, our scRNA-seq data did not reveal a substantial increase in immune cell populations, contrary to what has been reported in other studies^65–67^, while one study also did not show a decrease in the number of oocytes in aging ovaries based on their scRNA-seq data^67^. Hence, an adequate number of biological replicates are required to accurately represent all cell-type populations, considering that single-cell capture can be influenced by various factors, such as cell size and the preparation of single-cell samples. In addition, although we observed CD38 to be predominantly expressed in endothelial cells and immune cells, we did not elucidate whether CD38 regulates ovarian aging through their combined action or if one of these cell types predominantly influences it. Future work with cell-specific *Cd38*-knockout models will provide further insights into the mechanisms driving ovarian aging.

Taken together, our study describes the transcriptomic changes in early senescence of the ovary relative to other organs in middle-aged mice. At the molecular level, the early onset of inflammation-induced CD38 accumulation in middle-aged ovaries likely leads to a decrease in NAD^+^ level, accelerating cell senescence, disrupting transcriptional regulatory networks and altering cell–cell communication patterns in the ovary. These molecular changes ultimately result in reduced ovarian follicle reserve and compromised oocyte quality, contributing to age-related subfertility. Genetic deletion of CD38 attenuated ovarian aging and prolonged fertility in female mice. Importantly, our study demonstrates that CD38 plays a role in ovarian aging and may offer a valuable therapeutic approach to prolong ovarian lifespan, thereby mitigating the age-related decline in female fertility.

## Methods

This study received approval from the Ethics Committee of the First Affiliated Hospital of Zhengzhou University. Research with human biospecimens as well as animal studies were carried out in compliance with all relevant ethical regulations. Informed consent was obtained from the participants for the collection of follicular fluids.

### Reagents, antibodies and chemicals

Key resources in the form of reagents, antibodies and chemicals used in this study are shown in Supplementary Table 9.

### Animal feeding and treatments

Two-month-old and eight-month-old WT female C57BL/6 mice were obtained from Beijing Vital River Experimental Animals Center (Beijing, China). *Cd38*-knockout mice were generated by using CRISPR–Cas9 technology (Cyagen).

For genotyping of *Cd38*^−/−^ mice, PCR was performed on DNA extracted from mouse tails, and the *Cd38*-knockout mutant allele (509 bp) was assayed by the primers 5′-ACGGTTGGGACTTAGAACAGAG-3′ (forward) and 5′-TAGAAAGGGAAGCCCAGGTAAG -3′ (reverse). The *Cd38* WT allele (457 bp) was assayed by the primers 5′-TAGTGTCCAGTGCAGAGTATCTTC-3′ (forward) and 5′-GTAGAAAGGGAAGCCCAGGTAAG-3′ (reverse). All animals were housed in a pathogen-free environment in filter-top cages. For breeding experiments, WT, *Cd38*^−/−^ and CD38 inhibitor-treated females were individually caged with male C57BL/6 mice with proven fertility before monitoring the number of offspring. All mice were maintained under a 12-h light/dark cycle, at a room temperature of 20–25 °C, humidity of 55% ± 10%, and provided with food and water ad libitum.

### LPS treatment in vivo

For ovarian LPS treatments in vivo, 8-month-old female mice were injected intraperitoneally with LPS (Sigma) at a concentration of 1 mg ml^−1^ based on a previous study^68^, and then the ovaries were collected after 24 h for subsequent experiments.

### LPS treatment in vitro

For LPS treatment in vitro, ovaries from 10-day-old mice were placed on culture plate inserts (Millipore Corporation) and cultured in 400 µl of DMEM/F12 culture medium (Solarbio) supplemented with 10% FBS (Biological Industries) and 1% penicillin–streptomycin 100× solution (HyClone) in an incubator at 37 °C with 5% CO_2_ as previously reported^69^. A thin layer of medium covered the ovaries on the inserts. Ovaries were treated with 10 µg ml^−1^ LPS with media changes every 2 d for 4 d. At the end of the culture, ovaries were fixed in Bouin’s solution, paraffin-embedded and cut into continuous sections before staining with H&E. The growing and primordial follicle percentages were analyzed in these sections as previously reported^70^.

### CD38 inhibitor (78c) treatment in vivo

78c was administered intraperitoneally to 8-month-old female C57BL/6 mice at previously characterized doses of 15 mg per kg body weight twice daily for 8 d for NAD^+^ level measurement and 21 d for biological effects^60^. Control female mice received vehicle solutions with 10% dimethylsulfoxide, 40% PEG400, 5% Tween-80 and 45% PBS injections. Moreover, the FK866 (50 mg per kg body weight), an inhibitor for NAMPT, was used to treat the mice with or without 78c for 3 d, to verify the role of CD38 on ovarian NAD^+^ level regulation.

### Flow cytometry of human follicular fluid

Human follicular fluid was collected from young (20–25 years old) and middle-aged (>35 years old) participants who underwent an assisted reproductive technology procedure due to fallopian tube issues. Follicular fluid cells were isolated by density centrifugation at 850*g* for 15 min with Human Lymphocyte Separation Medium (Solarbio). After centrifugation, the intermediate layer was collected and centrifuged at 400*g* for 10 min. Then, cells were incubated in PBS labeled with the following antibody panel: CD38-APC (BioLegend) for 30 min at room temperature. After twice washing, samples were resuspended in PBS and subjected to a FACSMelody (BD Biosciences) for flow sorting. CD38-positive cells were extracted and frozen in TRIzol reagent for subsequent real-time RT–PCR experiments.

### IHC staining

Paraffin-embedded sections were initially deparaffinized with twice 100% xylene washes, rehydrated via graded alcohols and briefly washed in distilled water. The sections were treated using heat-mediated antigen retrieval with sodium citrate buffer (pH of 6.0) for 20 min, permeabilized and blocked in 1% BSA with 0.5% Triton X-100 for 1 h. The primary CD38 antibody was used at 4 °C overnight. After a wash with PBS, horseradish peroxidase-conjugated secondary antibodies were used before the 3,3′-diaminobenzidine application. The ovary sections were then counterstained with eosin and dehydrated in a series of graded alcohols and 100% xylene before they were coverslipped with resinous mounting medium.

### RNA isolation and quantitative real-time PCR

Total RNA was isolated from oocytes and other organs with TRIzol reagent (Invitrogen). cDNA was generated with HiScript III RT SuperMix for qPCR (+gDNA wiper; Vazyme). Quantitative real-time PCR was performed using ChamQ Universal SYBR qPCR Master Mix (Vazyme) on a QuantStudio 12K Flex (Applied Biosystems). The primer sequences are shown in Supplementary Table 10. Experiments were performed at least three times. Relative expression levels were determined by the delta CT method with normalization to *Gapdh*.

### Western blotting

Western blotting was performed as previously described^20^. Briefly, samples were sonicated in lysis buffer. After SDS‒PAGE, separated proteins were transferred to a PVDF transfer membrane (Millipore), and the membranes were incubated with specific primary and secondary antibodies sequentially. Protein bands were detected using an enhanced chemiluminescence detection system (Bio-Rad). ImageJ software was used to analyze protein expression levels after measuring the protein band intensity. All the antibodies used were validated by western blotting or IHC (Supplementary Fig. 3).

### NAD^+^ measurement

Ovarian NAD^+^ levels were measured using an NAD/NADH Assay Kit (Fluorometric; Abcam). Briefly, ovaries were collected and lysed in NAD/NADH lysis buffer before collecting the supernatant. Then, 25 ml of NAD^+^ extraction buffer was added to the lysate sample, followed by incubation at 37 °C for 15 min and the addition of 25 ml of NADH extraction buffer and 75 ml of NAD/NADH reaction mixture before incubation at room temperature for 1 h in the dark. Total NAD^+^ levels were quantified using a colorimetric assay at an excitation and emission of 540 and 590 nm, respectively, using a Varioskan Flash Multimode Reader (Thermo Fisher Scientific).

### Oocyte collection

Mice were superovulated by an intraperitoneal injection of 5 IU of pregnant mare serum gonadotropin, followed by 5 IU of human chorionic gonadotropin 48 h later. The mice were euthanized at 14 h after human chorionic gonadotropin injection. Cumulus–oocyte complexes were collected by dissecting oviductal ampullae in Gmops buffer (Vitrolife). Then, cumulus cells were removed by pipetting the cumulus–oocyte complexes in 0.1% hyaluronidase (Solarbio) in Gmops.

### ROS level monitoring

For measurement of the ROS content, MII oocytes were incubated in IVF medium with 5 mM MitoSOX Red reagent prepared according to the instructions of the MitoSOX Red mitochondrial superoxide indicator (Thermo Fisher Scientific) at 37 °C for 20 min in a 5% CO_2_ incubator. After washing three times with IVF medium, oocyte images were captured under a laser scanning confocal microscope (LSM 700; Zeiss). The fluorescence value was calculated using ImageJ software (National Institutes of Health).

### Mitochondrial membrane potential (Δψm) measurement

Oocytes were incubated with 10 µM JC-1 (Beyotime) at 37 °C in 5% CO_2_ for 20 min in the dark. After three washes in PBS, oocyte images were captured in both the green and red channels using an LSM 700 laser scanning confocal microscope. Fluorescence intensities were detected using ImageJ. The ΔΨm was calculated as the ratio of red-to-green fluorescence pixels.

### Spindle assembly analysis

MII oocytes were obtained and fixed in PBS (Solarbio) containing 4% paraformaldehyde for 30 min, followed by permeabilization in PBS with 0.5% Triton X-100 for 20 min. After blocking with 1% BSA in PBS for 30 min, oocytes were incubated with an anti-α-tubulin antibody (Cell Signaling Technology) overnight at 4 °C. After three washes, oocytes were incubated with a FITC-conjugated secondary Alexa Fluor 488 antibody (Thermo Fisher Scientific) for 1 h at room temperature. For spindle assembly analysis, oocytes were stained with propidium iodide and then viewed under an LSM 700 laser scanning confocal microscope.

### Sectioning ovaries for follicle counting and serum AMH measurement

Ovaries were fixed with 4% formaldehyde for 24 h, washed with PBS two times and stored in 70% ethanol. After embedding in paraffin wax and serial sectioning at a thickness of 5 μm, ovarian sections were deparaffinized and rehydrated through a graded ethanol series. The samples were then stained with H&E. Quantification of ovarian follicles in different developmental stages, including primordial, primary, secondary, antral and atretic follicles, was performed as previously described^69^. Blood samples were drawn via the retro-orbital sinus, and serum samples were extracted and kept at −80 °C until use. Serum AMH measurements were performed using an enzyme-linked immunosorbent assay (ELISA) according to the manufacturer’s instructions (CUSABIO).

### TUNEL assay and immunofluorescence on ovarian sections

Paraffin-embedded ovarian sections were heated for 45 min at 65 °C for deparaffinization, and the slides were then treated with 100% xylene three times followed by washing three times with 100% ethanol. The slides were rehydrated with citrate buffer (pH 6.0) for 20 min, permeabilized and blocked in 1% BSA with 0.5% Triton X-100 for 1 h. The TUNEL assay was conducted according to the manufacturer’s protocol (Roche, Swiss). For immunofluorescence, the slides were incubated with primary antibodies against γH2AX (1:100 dilution) overnight at 4 °C, followed by incubation with secondary antibodies conjugated with Alexa Fluor 555 (Thermo Fisher Scientific; 1:200 dilution) and then with Antifade Mounting Medium with DAPI. Images were captured using a fluorescence microscope (Ni2 Nikon).

### PSR staining

The PSR Stain Kit (Polysciences) was used following the manufacturer’s protocol. Briefly, ovary sections underwent deparaffinization with xylene and ethanol gradient, followed by water washes. Subsequently, sections were treated with solution A for 2 min and solution B for 1 h at room temperature, and slides were cleared using solution C. For each separate experiment, all PSR-stained slides were processed simultaneously to reduce staining intensity variation. Fibrotic area in imaged ovaries stained with PSR was measured using ImageJ.

### Measurement of CD38 enzymatic activities

CD38 activities were measured according to the manufacturer’s protocol (CUSABIO). Briefly, tissues were washed with PBS, homogenized in 1 ml of PBS and stored overnight at −20 °C. After two freeze–thaw cycles to break cell membranes, the homogenates were centrifuged for 5 min. The supernatant was removed and assayed immediately, and the CD38 activity was calculated.

### Tissue RNA-seq data processing

For gene expression quantification, uniquely mapped reads were submitted as reads per kilobase of exon per million reads mapped for the gene algorithm to calculate counts of fragments per kilobase of exon and per million mapped reads (FPKMs). Samples from different groups were initially analyzed by PCA based on FPKM values of the expression to characterize the trend of intergroup separation in the experimental model. The PCA plots and differential gene expression were analyzed with the R package DESeq2. Genes with |log_2_FC| > 1 and adjusted *P* value (by the Benjamini–Hochberg method) < 0.05 were considered DEGs. Heat maps generated by the R package pheatmap were used to visualize the expression patterns of DEGs in different groups. The DEGs were further divided into upregulated and downregulated genes according to log_2_FC for enrichment analysis. GO and Kyoto Encyclopedia of Genes and Genomes (KEGG) analyses were performed by the R package clusterProfiler. GO terms and KEGG pathways with a *P* value < 0.05 were considered statistically significant and are shown by dot plots. *P* values were calculated by Fisher’s exact test and adjusted by the Benjamini–Hochberg method. To compare the differences in biological process signaling pathways between different groups, GSEA was also used. GSEA analysis was performed by the R package clusterProfiler, and *P* values were calculated based on Fisher’s exact test. Only gene sets with *P* value < 0.05 were shown by using the R package enrichplot.

### Single-cell RNA-seq library construction and sequencing

Ovaries were washed in ice-cold RPMI 1640 and dissociated using collagenase IV, Dispase and DNase I. After cell counting, fresh cells were washed twice in the RPMI 1640 and then resuspended at 1 × 10^6^ cells per ml in PBS with 0.04% BSA. Then, the single-cell RNA-seq libraries were prepared using SeekOne Digital Droplet Single Cell 3′ library preparation kit (SeekGene) and libraries were sequenced on an Illumina NovaSeq 6000 with PE150 read length.

### Analysis of scRNA-seq data of ovaries

The raw sequencing data were processed by Fastp firstly to trim primer sequence and eliminate low-quality bases. Subsequently, SeekOneTools was used to preprocess sequencing data and align to GRCm38 in order to obtain gene expression matrix. For further analysis of scRNA-seq data, R package Seurat (version 4.3.0.1)^71^ and Harmony (version 0.1.1)^72^ were used for filtering, data normalization, dimensionality reduction, clustering and further analysis of the single-cell RNA-seq data. Briefly, data from three groups of ovaries (WT-2M, WT-12M and *Cd38*^−/−^-12M) were combined by Harmony package and were clustered to 22 cell types. Then the clusters were annotated using cell-type-specific signatures and marker genes^73,74^ into six cell types: granulosa cells, stromal cells, immune cells, endothelial cells, epithelial cells and oocytes. Gene-set scores of ‘SASP’, ‘DNA repair’ and ‘cell cycle’ for each input cell were calculated using the Seurat function ‘AddModuleScore’. Changes in the scores in the ‘aging group’ (WT-12M versus WT-2M) or ‘*Cd38*^−/−^ group’ (*Cd38*^−/−^-12M versus WT-12M) were analyzed by the Wilcoxon test. Then differential gene expression analysis was performed with the ‘FindMarkers’ function of Seurat in the ‘aging group’ and ‘*Cd38*^−/−^ group’. DEGs were defined as those genes with *P* values < 0.05 and |log_2_FC| > 0.25. GO enrichment was performed on DEGs in each cell type.

Then TF regulatory network analysis was conducted using pySCENIC (version 0.10.4) with default settings^75^. The transcriptional regulatory network was visualized by Cytoscape (version 3.9.1). CellPhoneDB Python package (version 2.1.7)^76^ was used to detect ligand–receptor interactions and predict communications among different cell types. Only those specific interactions classified by ligand–receptor expression in more than 10% of cells within a cell type were selected. Average expression of each ligand–receptor pair was compared across cell types, and only those with a significance level of *P* < 0.05 were used for subsequent cell–cell communication prediction.

### Single-oocyte RNA-seq library construction and sequencing

Mouse MII oocytes were obtained as described above. A single MII oocyte was transferred to a PCR tube with lysis buffer containing dNTP and oligo (dT) oligonucleotides. Then, a sequencing library from a single oocyte was constructed using a SMART-seq HT Kit (Takara). Purified libraries were quantified and validated by a Qubit 3.0 Fluorometer and Agilent 2100 Bioanalyzer to confirm the insert size and calculate the concentration. Then, sequencing was performed on an Illumina NovaSeq 6000 (Illumina). RNA-seq reads were aligned to the GRCm38.100 reference genome using Hisat2 software. For subsequent downstream analysis, the original expression reads were converted to FPKMs according to the method described above.

### Single-oocyte RNA-seq data processing and pathway analysis

The single-cell sequencing data analysis was similar to that described for the tissue RNA-seq data above. The PCA plot for the three groups was drawn, and then DEGs of two groups were calculated by using data from 12-month-old versus 2-month-old WT mice and 12-month-old *Cd38*^−/−^ versus 12-month-old WT mice. The expression patterns of the DEGs are shown in a heat map. Next, the intersecting genes among the downregulated DEGs due to aging and upregulated DEGs due to the knockout assay were selected. GO and KEGG analyses were performed to explore the functional enrichment of these intersecting genes. All the pathway-related genes mentioned in this paper were obtained by the combination of related terms from the GO database. The gene list is shown in Supplementary Table 11.

### Statistics and reproducibility

We did not use a statistical method to determine the sample size in advance, but we consistently used similar sample sizes for each experiment. Percentages or values are shown as the mean ± s.e.m. Statistical significance was measured using Student’s *t*-test, chi-squared test with Prism software (GraphPad). Values of *P* < 0.05 were considered statistically significant. The specific sample sizes (the *n* numbers) are provided in the figure legends. In addition, for all experiments except RNA-seq data, each experiment was conducted independently at least three times. The statistical test methods and exact *P* values are detailed in the corresponding figure legends. No specific methods were used for random allocation of samples into groups. All data were included in the analysis without any exclusions. Data collection and analysis were conducted without blinding to the experimental conditions. Although we assumed a normal data distribution with equal variance, this assumption was not formally tested.

### Reporting summary

Further information on research design is available in the Nature Portfolio Reporting Summary linked to this article.

### Supplementary information

Supplementary Information Legends for Supplementary Tables 1–11, Supplementary Figs. 1–3 and image source data for Supplementary Fig. 3.

Reporting Summary

Supplementary Table Supplementary Table 1. RNA-seq data of different tissues. Supplementary Table 2. Basic clinical characteristics of young and middle-aged participants. RNA-seq expression data between *Cd38*-knockout and WT mice in 8-month-old ovaries. Supplementary Table 3. RNA-seq expression data between *Cd38*-knockout and WT in 8-month-old ovaries. Supplementary Table 4. RNA-seq expression data of *Cd38*-knockout and WT in 12-month-old ovaries. Supplementary Table 5. Specific markers used to annotate cell clusters. Supplementary Table 6. Single-oocyte sequencing data showing the *Cd38*-knockout and WT at 2 months and 12 months old. Supplementary Table 7. The overlap genes between age-related downregulated (aged versus young) and upregulated after deletion of CD38. Supplementary Table 8. Mitochondrial-related gene list. Supplementary Table 9. Reagents, antibodies and chemicals. Supplementary Table 10. Real time RT–PCR primer sequences. Supplementary Table 11. Pathway genelists include SASP, inflammation, DNA repair and cell cycle. Statistical Source Data for Supplementary Fig. 2

### Source data

Source Data Fig. 1 Statistical source data.

Source Data Fig. 1 Unprocessed western blots.

Source Data Fig. 2 Statistical source data.

Source Data Fig. 2 Unprocessed western blots.

Source Data Fig. 3 Statistical source data.

Source Data Fig. 6 Statistical source data.

Source Data Fig. 7 Statistical source data.

Source Data Fig. 7 Unprocessed western blots.

Source Data Fig. 8 Statistical source data.

Source Data Extended Data Fig. 2 Statistical source data.

Source Data Extended Data Fig. 2 Unprocessed western blots.

Source Data Extended Data Fig. 6 Statistical source data.

## Data Availability

The raw data of scRNA-seq and bulk RNA-seq presented in this study have been deposited into the Sequence Read Archive database under the accession number PRJNA1002222. Source data are provided with this paper. Any other data underlying this study will be provided by the corresponding authors upon reasonable request.

## Code availability

The software used in this study is publicly available and accessed without restriction. R package DESeq2 v.1.34.0 is available at https://www.bioconductor.org/packages/release/bioc/html/DESeq2.html. R package clusterProfiler v.4.2.0 is available at https://www.bioconductor.org/packages/release/bioc/html/clusterProfiler.html. R package pheatmap v.1.0.12 is available at https://rdocumentation.org/packages/pheatmap/versions/1.0.2. R package enrichplot v.1.14.1 is available at https://www.bioconductor.org/packages/release/bioc/html/enrichplot.html. R package GSEABase v.1.56.0 is available at https://www.bioconductor.org/packages/release/bioc/html/GSEABase.html. R package ggplot2 v.3.3.6 is available at https://cran.r-project.org/web/packages/ggplot2/index.html. R package stringr v.1.4.0 is available at https://cran.r-project.org/web/packages/stringr/index.html. R package tidyverse v.1.3.1 is available at https://cran.r-project.org/web/packages/tidyverse/index.html. R package VennDiagram v.1.7.1 is available at https://cran.r-project.org/web/packages/VennDiagram/index.html. R package Seurat v.4.3.0.1 is available at https://github.com/satijalab/seurat/. Harmony v.0.1.1 is available at https://cran.r-project.org/web/packages/harmony/index.html. R package AUCell v.1.22.0 is available at https://www.bioconductor.org/packages/release/bioc/html/AUCell.html. Python package pySCENIC v.0.12.0 is available at https://github.com/aertslab/pySCENIC/. For analysis of cell–cell interaction, CellPhoneDB v.3.1.0 is available at https://github.com/ventolab/CellphoneDB/. R Studio v.4.1.2 and Python v.3.7.12 were used to format data for analyses.
