# Supplementary material for: NADase CD38 is a key determinant of ovarian aging
Source: Nat Aging. 2023 Dec 21;4(1):110–28. doi: 10.1038/s43587-023-00532-9 (PMC10798903; doi:10.1038/s43587-023-00532-9)
Supplement: Supplementary file 1 — Legends for Supplementary Tables 1–11, Supplementary Figs. 1–3 and image source data for Supplementary Fig. 3. [file 43587_2023_532_MOESM1_ESM.pdf]

nature aging

Article https://doi.org/10.1038/s43587-023-00532-9

# NADase CD38 is a key determinant of ovarian aging

In the format provided by the authors and unedited

Supplementary Table 1

RNA-Seq data of different tissues. The *P* values were calculated by the Wald test and the padj was corrected for multiple testing using the Benjamini and Hochberg method.

Supplementary Table 2

Basic clinical characteristics of young and middle-aged patients. Data are presented as the mean ± SEM. *P* value was determined by the unpaired *t*-test, two-tailed between each two groups. **P*< 0.05, ***P*< 0.01, ****P*< 0.001.

Supplementary Table 3

RNA-Seq expression data between *Cd38* Knock-out and WT in 8M ovaries. The *P* values were calculated by the Wald test and the padj was corrected for multiple testing using the Benjamini and Hochberg method.

Supplementary Table 4

RNA-Seq expression data of *Cd38* Knock-out and WT at age of 12-month-old ovaries. The *P* values were calculated by the Wald test and the padj was corrected for multiple testing using the Benjamini and Hochberg method.

Supplementary Table 5

Specific markers used to annotate cell clusters.

Supplementary Table 6

Single-oocyte sequencing data showing the *Cd38* Knockout and WT at ages of 2- and 12-month-old. The *P* values were calculated by the Wald test and the padj was corrected for multiple testing using the Benjamini and Hochberg method.

Supplementary Table 7

The overlap genes between age-related downregulated (Aged vs. Young) and upregulated after deletion of CD38.

Supplementary Table 8

Mitochondrial-related gene list.

Supplementary Table 9

Reagents, antibodies, and chemicals.

Supplementary Table 10

Real time RT- PCR primer sequences.

Supplementary Table 11

Pathway genelists include SASP, inflammation, DNA repair, and cell cycle.

Statistical Source Data Supplementary Fig. 2

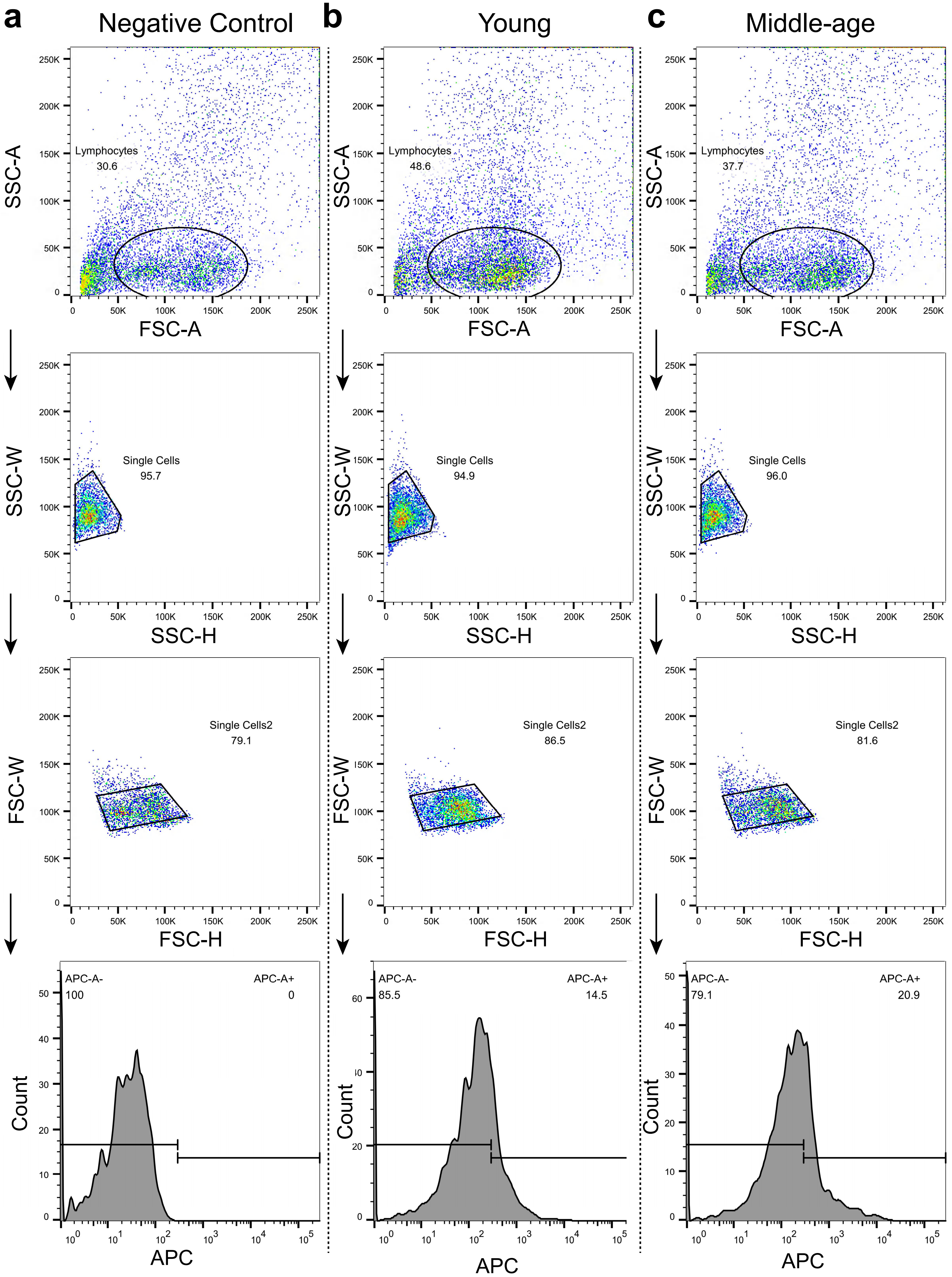

a
Negative Control
b
Young
c
Middle-age
Lymphocytes
30.6
Lymphocytes
48.6
Lymphocytes
37.7
SSC-A
FSC-A
Single Cells
95.7
Single Cells
94.9
Single Cells
96.0
SSC-W
SSC-H
Single Cells2
79.1
Single Cells2
86.5
Single Cells2
81.6
FSC-W
FSC-H
APC-A-
100
APC-A+
0
APC-A-
85.5
APC-A+
14.5
APC-A-
79.1
APC-A+
20.9
Count
APC

**Supplementary Fig. 1. Gating strategies used for CD38 positive cell sorting from follicular fluids.**

a. Gating strategy for follicular fluids without fluorescent dye (PBS) as a negative control. First, by setting the coordinate axes to FSC-A and SSC-A, different cell types were distinguished, and a gate was applied to the target cells and filter out cell fragments. Then, the setting of the horizontal and vertical axes as SSC-H, SSC-W, and FSC-H, FSC-W was performed to remove cell adhesion or aggregates. Finally, in the form of histograms, a gate was applied in the negative control group, with the leftside representing negative and the right side representing positive cells. The same strategy was applied to both the follicular fluids of young- (b) and middle-aged patients (c) labeled with the following antibody panel: CD38-APC. The proportion of APC-positive cells to the right of the gate was observed between the two groups.

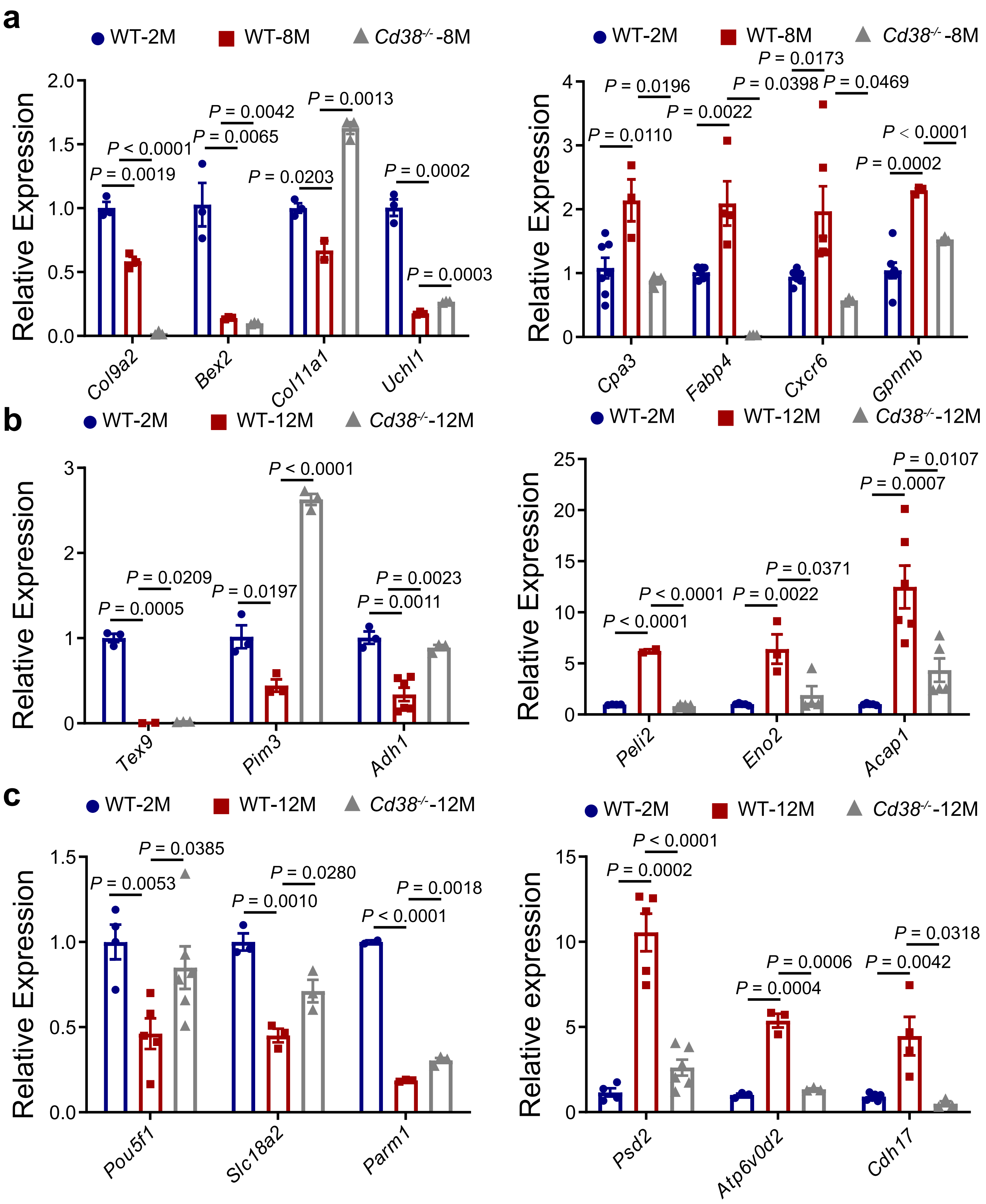
a
WT-2M
WT-8M
Cd38-/- -8M
Relative Expression
P = 0.0019
P < 0.0001
P = 0.0065
P = 0.0042
P = 0.0203
P = 0.0013
P = 0.0002
P = 0.0003
Col9a2
Bex2
Col11a1
Uchl1
P = 0.0110
P = 0.0196
P = 0.0022
P = 0.0398
P = 0.0173
P = 0.0469
P = 0.0002
P < 0.0001
Cpa3
Fabp4
Cxcr6
Gpnmb
b
WT-2M
WT-12M
Cd38-/- -12M
P = 0.0005
P = 0.0209
P = 0.0197
P < 0.0001
P = 0.0011
P = 0.0023
Tex9
Pim3
Adh1
P < 0.0001
P < 0.0001
P = 0.0022
P = 0.0371
P = 0.0007
P = 0.0107
Peli2
Eno2
Acap1
c
P = 0.0053
P = 0.0385
P = 0.0010
P = 0.0280
P < 0.0001
P = 0.0018
Pou5f1
Slc18a2
Parm1
Relative expression
P = 0.0002
P < 0.0001
P = 0.0004
P = 0.0006
P = 0.0042
P = 0.0318
Psd2
Atp6v0d2
Cdh17

**Supplementary Fig. 2. Real time RT-PCR validation of up- and down-regulated DEGs in middle-aged (8-month-old) mice.**

a. Randomly selected down- (left panel) and up-regulated (right panel) DEGs expression validation in 2-, 8-month-old WT and *Cd38*$^{-/-}$ ovaries (n= 3-7 for each group).

b. Randomly selected down- (left panel) and up-regulated (right panel) DEGs expression validation in 2-, 12-month-old WT and *Cd38*$^{-/-}$ ovaries (n= 3-6 for each group).

c. Randomly selected down- (left panel) and up-regulated (right panel) DEGs expression validation in 2-, 12-month-old WT and *Cd38*$^{-/-}$ oocytes (n= 3-6 for each group). Data are represented as mean ± s.e.m. *P* value was determined by the unpaired *t*-test, two-tailed.

Validated of used antibodies

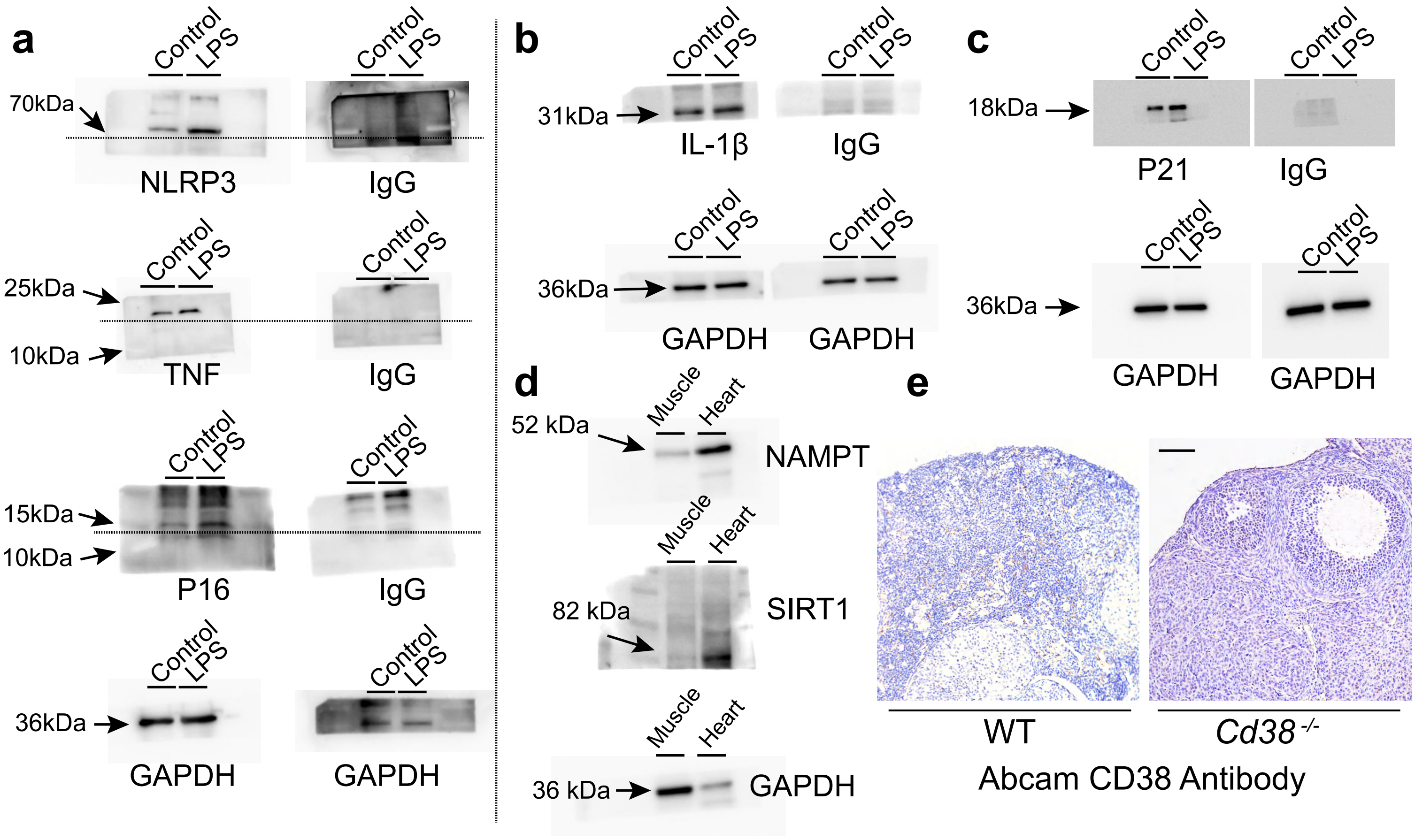

**Supplementary Fig. 3. Verification of antibodies used in this study.**

All employed antibodies were validated. For NLRP3, TNF, P16 (a), IL-1β(b), and P21 (c), KGN cells treated with or without 10 μg/ml LPS in DMEM-F12 medium for 60h were used to validate the antibody specificity. Negative controls used IgG corresponding to the respective antibody's reactivity. All proteins were probed alongside the internal control GAPDH. The results showed increased expression of the mentioned proteins after LPS treatment, and the IgG control exhibited no bands at the corresponding protein positions. For NAMPT and SIRT1 (d), we validated the antibodies by observing their expression in muscle and heart tissues. Regarding the CD38 antibody (e), the CD38 antibody from Abcam was used, and its validation was performed on ovarian tissue slices from WT and *Cd38*$^{-/-}$ mice by IHC. Scale bar, 100 μm.

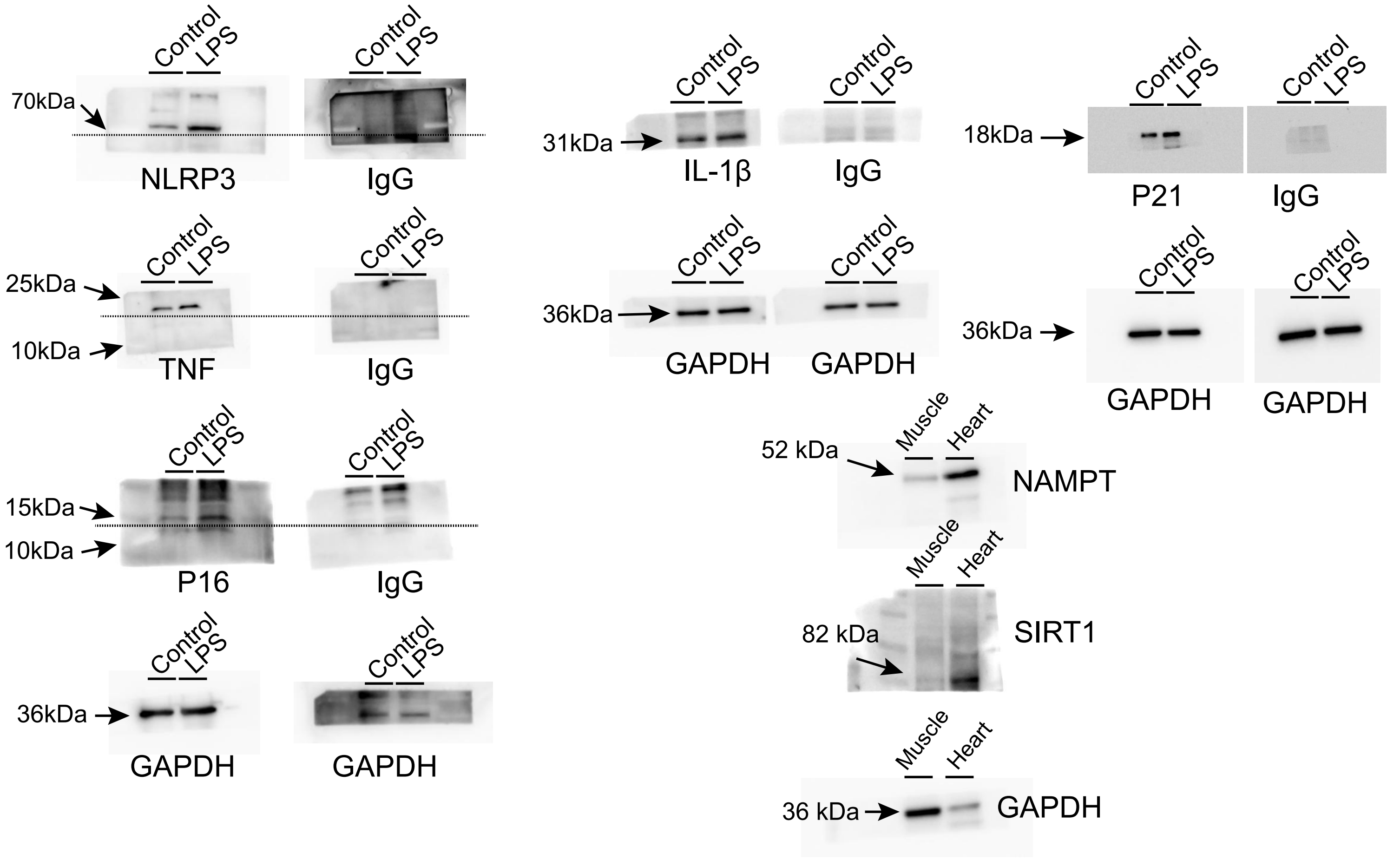


**Source Data for Supplementary Fig. 3.** Unprocessed western blots.
