# Supplementary material for: NADase CD38 is a key determinant of ovarian aging
Source: Nat Aging. 2023 Dec 21;4(1):110–28. doi: 10.1038/s43587-023-00532-9 (PMC10798903; doi:10.1038/s43587-023-00532-9)
Supplement: Supplementary file 2 — Reporting Summary [file 43587_2023_532_MOESM2_ESM.pdf]

nature portfolio

Corresponding author(s): Qingling Yang, and Yingpu Sun

Last updated by author(s): Nov 25, 2023

Reporting Summary

Nature Portfolio wishes to improve the reproducibility of the work that we publish. This form provides structure for consistency and transparency in reporting. For further information on Nature Portfolio policies, see our Editorial Policies and the Editorial Policy Checklist.

Statistics

For all statistical analyses, confirm that the following items are present in the figure legend, table legend, main text, or Methods section.

n/a	Confirmed	
☐	☒	The exact sample size (*n*) for each experimental group/condition, given as a discrete number and unit of measurement
☐	☒	A statement on whether measurements were taken from distinct samples or whether the same sample was measured repeatedly
☐	☒	The statistical test(s) used AND whether they are one- or two-sided<br>*Only common tests should be described solely by name; describe more complex techniques in the Methods section.*
☐	☒	A description of all covariates tested
☐	☒	A description of any assumptions or corrections, such as tests of normality and adjustment for multiple comparisons
☐	☒	A full description of the statistical parameters including central tendency (e.g. means) or other basic estimates (e.g. regression coefficient) AND variation (e.g. standard deviation) or associated estimates of uncertainty (e.g. confidence intervals)
☐	☒	For null hypothesis testing, the test statistic (e.g. *F*, *t*, *r*) with confidence intervals, effect sizes, degrees of freedom and *P* value noted<br>*Give P values as exact values whenever suitable.*
☒	☐	For Bayesian analysis, information on the choice of priors and Markov chain Monte Carlo settings
☒	☐	For hierarchical and complex designs, identification of the appropriate level for tests and full reporting of outcomes
☒	☐	Estimates of effect sizes (e.g. Cohen's *d*, Pearson's *r*), indicating how they were calculated

Our web collection on statistics for biologists contains articles on many of the points above.

Software and code

Policy information about availability of computer code

Data collection	Image Lab, Bio-Rad<br>ZEISS LSM700- LASER SCANNING CONFOCAL, Zeiss<br>Stereoscope SMZ800N, Nikon<br>QuantStudio 12K Flex Real-Time PCR System, Applied Biosystems<br>DS-Qi2, Nikon<br>NanoDrop ND-2000, Thermo Fisher Scientific<br>FACSMelody, BD biosciences
Data analysis	Image J (bundled with Java 8), NIH, (image processiong/analysis)<br>GraphPad Prism 9, GraphPad Software (statistical analysis)<br>IBM SPSS Statistics 26.0, IBM (statistical analysis), flowjo (version 10.8.1),<br>R (version 4.3.0), R Studio (Version 4.1.2) (RNA-Seq data analysis), R packages used were as follows: DESeq2 (version: 1.34.0), clusterProfiler(version 4.2.0), pheatmap(1.0.12), enrichplot(1.14.1), ggord(1.1.6), GSEABase(1.56.0), ggplot2(3.3.6), stringr(1.4.0), tidyverse(1.3.1), VennDiagram(1.7.1)， Seurat (version 4.3.0.1), Fastp (version 0.23.1), Harmony (version 0.1.1), Python (version 3.7.12), pySCENIC(version 0.10.4),CellPhoneDB(version 2.1.7).

For manuscripts utilizing custom algorithms or software that are central to the research but not yet described in published literature, software must be made available to editors and reviewers. We strongly encourage code deposition in a community repository (e.g. GitHub). See the Nature Portfolio guidelines for submitting code & software for further information.

Data

Policy information about availability of data

All manuscripts must include a data availability statement. This statement should provide the following information, where applicable:

- Accession codes, unique identifiers, or web links for publicly available datasets
- A description of any restrictions on data availability
- For clinical datasets or third party data, please ensure that the statement adheres to our policy

All data needed to evaluate the conclusions in the paper are present in the paper and/or the Supplementary Materials. The raw data of ScRNA-seq and bulk RNA-seq presented in this study have been deposited into the Sequence Read Archive database under the accession number PRJNA1002222 and have been released. Additional data that support the findings of this study are available on request from the corresponding author.

Human research participants

Policy information about studies involving human research participants and Sex and Gender in Research.

Reporting on sex and gender	This study included female mice, male mice and infertility female were included.
Population characteristics	Human follicular fluid was collected from young (20–25 years old, n=5) and middle-aged (> 35 years old, n=15) patients who underwent an assisted reproductive technology procedure due to fallopian tube issues.
Recruitment	Follicular fluids were collected from young (20-25 years old) and middle-aged (> 35 years old) patients with blocked fallopian tubes. We collect patient samples that meet the recruitment criteria within a specific time period without any patient selection to avoid introducing any artificial factors that could affect the experimental results.
Ethics oversight	The collection of follicular fluid and the separation of granulosa cells of young and middle-aged infertile female were approved by the Ethics Committee of the First Affiliated Hospital of Zhengzhou University.

Note that full information on the approval of the study protocol must also be provided in the manuscript.

Field-specific reporting

Please select the one below that is the best fit for your research. If you are not sure, read the appropriate sections before making your selection.

☒ Life sciences ☐ Behavioural & social sciences ☐ Ecological, evolutionary & environmental sciences

For a reference copy of the document with all sections, see nature.com/documents/nr-reporting-summary-flat.pdf

Life sciences study design

All studies must disclose on these points even when the disclosure is negative.

Sample size	We did not employ a statistical method to determine the sample size in advance, but we consistently used similar sample sizes for each experiment. The exact sample sizes (i.e., the n numbers) are indicated in the figure legends.
Data exclusions	There was no data excluded in this study.
Replication	Each experiment was repeated at least three times, with multiple repetitions for verification as shown in figure legends.
Randomization	No randomization method was used to allocate animals experiments, as these were performed on already randomized animals in each condition. Infertility patients were allocated to groups based on their age.
Blinding	Data collection and analysis were conducted without blinding to the experimental conditions. Blinding was not incorporated into our experimental design as we employed multiple biological replicates and independent experiments to ensure reproducibility.

Reporting for specific materials, systems and methods

We require information from authors about some types of materials, experimental systems and methods used in many studies. Here, indicate whether each material, system or method listed is relevant to your study. If you are not sure if a list item applies to your research, read the appropriate section before selecting a response.

Materials & experimental systems

n/a	Involved in the study
☐	☒ Antibodies
☒	☐ Eukaryotic cell lines
☒	☐ Palaeontology and archaeology
☐	☒ Animals and other organisms
☒	☐ Clinical data
☒	☐ Dual use research of concern

Methods

n/a	Involved in the study
☒	☐ ChIP-seq
☐	☒ Flow cytometry
☒	☐ MRI-based neuroimaging

Antibodies

Antibodies used

Rabbit anti-P16 antibody, Abcam, Cambridge, UK, Cat#ab211542, 1:1000 dilution.
Rabbit anti-P21 antibody, Abcam, Cambridge, UK, Cat#ab188224, 1:1000 dilution.
Rabbit anti-CD38 antibody Abcam, Cambridge, UK, Cat# ab216343, 1:1000 dilution.
Mouse anti-CD38 antibody, R&D systems, MN, USA, Cat# AF4947, 1:400 dilution.
Mouse anti-IL-1β antibody, Proteintech, WuHan, China, Cat#16806-1-AP, 1:1000 dilution.
Rabbit anti-NLRP3 antibody, Proteintech, WuHan, China, Cat#19771-1-AP, 1:1000 dilution.
TNF Alpha Monoclonal antibody, Proteintech, WuHan, China, Cat#60291-1-Ig, 1:1000 dilution.
Mouse anti-SIRT1 antibody, Proteintech, WuHan, China, Cat#60303-1-Ig, 1:1000 dilution.
Rabbit anti-NAMPT antibody, Affinity Biosciences, Ancaster, Canada, Cat# DF6059, 1:1000 dilution.
Mouse anti-γH2A.X antibody, Cell Signaling Technology, MA, USA, Cat# 80312, 1:100 dilution.
Rabbit anti-α-Tubulin antibody, Cell Signaling Technology, MA, USA, Cat# 5335, 1:150 dilution.
Rabbit anti-GAPDH antibody, Proteintech, WuHan, China, Cat# 10494-1-AP, 1:2000 dilution.
Mouse anti-FOXO3A antibody, Proteintech, WuHan, China, Cat# 66428-1-Ig, 1:1000 dilution.
HRP-conjugated Affinipure Goat Anti-Rabbit IgG(H+L), Proteintech, WuHan, China, Cat# SA00001-2, 1:2000 dilution.
HRP-conjugated Affinipure Goat Anti-Mouse IgG(H+L), Proteintech, WuHan, China, Cat# SA00001-1, 1:2000 dilution.
Donkey anti-Rabbit IgG (H+L) Highly Cross-Adsorbed Secondary Antibody, Alexa Fluor™ 488, Thermo Fisher Scientific, MA, USA, Cat# A-21206, 1:200 dilution.
Donkey anti-Mouse IgG (H+L) Highly Cross-Adsorbed Secondary Antibody, Alexa Fluor™ 555, Thermo Fisher Scientific, MA, USA, Cat# A-31570, 1:200 dilution.
APC anti-human CD38 antibody, BioLegend, CA, USA, Cat#303509,1:100 dilution.

Validation

The antibodies utilized in this study are all commercially available and have been validated. The validation of these antibodies is presented in Extended Data Fig.9. In brief, we validated the antibodies for NLRP3, TNF, P16, and P21 by using KGN cells treated with or without 10 μg/ml LPS in DMEM-F12 medium for 60 hours. Negative controls involved using IgG corresponding to the reactivity of the respective antibodies. For NAMPT and SIRT1, antibody validation was carried out by observing their expression in muscle and heart tissues. CD38 antibodies were validated through Western blot and immunohistochemistry (IHC) in WT and Cd38-/- mice.

Animals and other research organisms

Policy information about studies involving animals; ARRIVE guidelines recommended for reporting animal research, and Sex and Gender in Research

Laboratory animals

Two-month-old, eight-month-old, and twelve-month-old wild-type C57/BL6 mice were used in this study were from Beijing Vital River Experimental Animals Centre (Beijing, China). The Cd38 knockout mouse was generated by Crisper/Cas9 (Cyagen, Hangzhou, China). All animals were housed in a pathogen-free environment in filter-top cages. All mice were maintained under a 12-h light/darkness cycle, room temperature between 20 - 25°C, humidity of 55% ± 10, and provided with food and water ad libitum.

Wild animals

No wild animals were used in this study.

Reporting on sex

The female mice were used to investigate ovarian functions and oocyte quality. The number of female mice used for each experiment was listed in the figure legends. For breeding experiments in this study, 2-month-old WT, Cd38−/− and CD38 inhibitor-treated females were individually caged with male C57/BL6 mice with proven fertility for a mating period of 1 month.

Field-collected samples

No field-collected animals were used in this study.

Ethics oversight

All of the experimental procedures followed the guidelines of NIH for the Care and Use of Laboratory Animals. This study obtained approval from the Ethics Committee of the First Affiliated Hospital of Zhengzhou University.

Note that full information on the approval of the study protocol must also be provided in the manuscript.

Flow Cytometry

Plots

Confirm that:

☒ The axis labels state the marker and fluorochrome used (e.g. CD4-FITC).

☒ The axis scales are clearly visible. Include numbers along axes only for bottom left plot of group (a 'group' is an analysis of identical markers).

☒ All plots are contour plots with outliers or pseudocolor plots.

☒ A numerical value for number of cells or percentage (with statistics) is provided.

Methodology

Sample preparation	Follicular fluid cells were isolated by density centrifugation at 850 × g for 15 min with Human Lymphocyte Separation Medium (Solarbio, Beijing, China). After centrifugation, the intermediate layer was collected and centrifuged at 400 × g for 10 min. Then, Cells were incubated in PBS labeled with the following antibody panel: CD38-APC (BioLegend, CA, USA) for 30 min at room temperature. After twice washing, samples were resuspended in PBS and subjected to a FACSMelody (BD Biosciences, NJ, USA) for flow sorting.
Instrument	FACSMelody, BD biosciences
Software	FACSMelody, data were analyzed in flowjo v.10.8.1
Cell population abundance	CD38 positive cells
Gating strategy	First, by setting the coordinate axes to FSC-A and SSC-A, different cell types were distinguished, and a gate was applied to the target cells and filter out cell fragments. Then, the setting of the horizontal and vertical axes as SSC-H, SSC-W, and FSC-H, FSC-W was performed to remove cell adhesion or aggregates. Finally, in the form of histograms, a gate was applied in the negative control group, with the left side representing negative and the right side representing positive cells.

☒ Tick this box to confirm that a figure exemplifying the gating strategy is provided in the Supplementary Information.
