# Supplementary figures and images for: NADase CD38 is a key determinant of ovarian aging
Source: Nat Aging. 2023 Dec 21;4(1):110–28. doi: 10.1038/s43587-023-00532-9 (PMC10798903; doi:10.1038/s43587-023-00532-9)

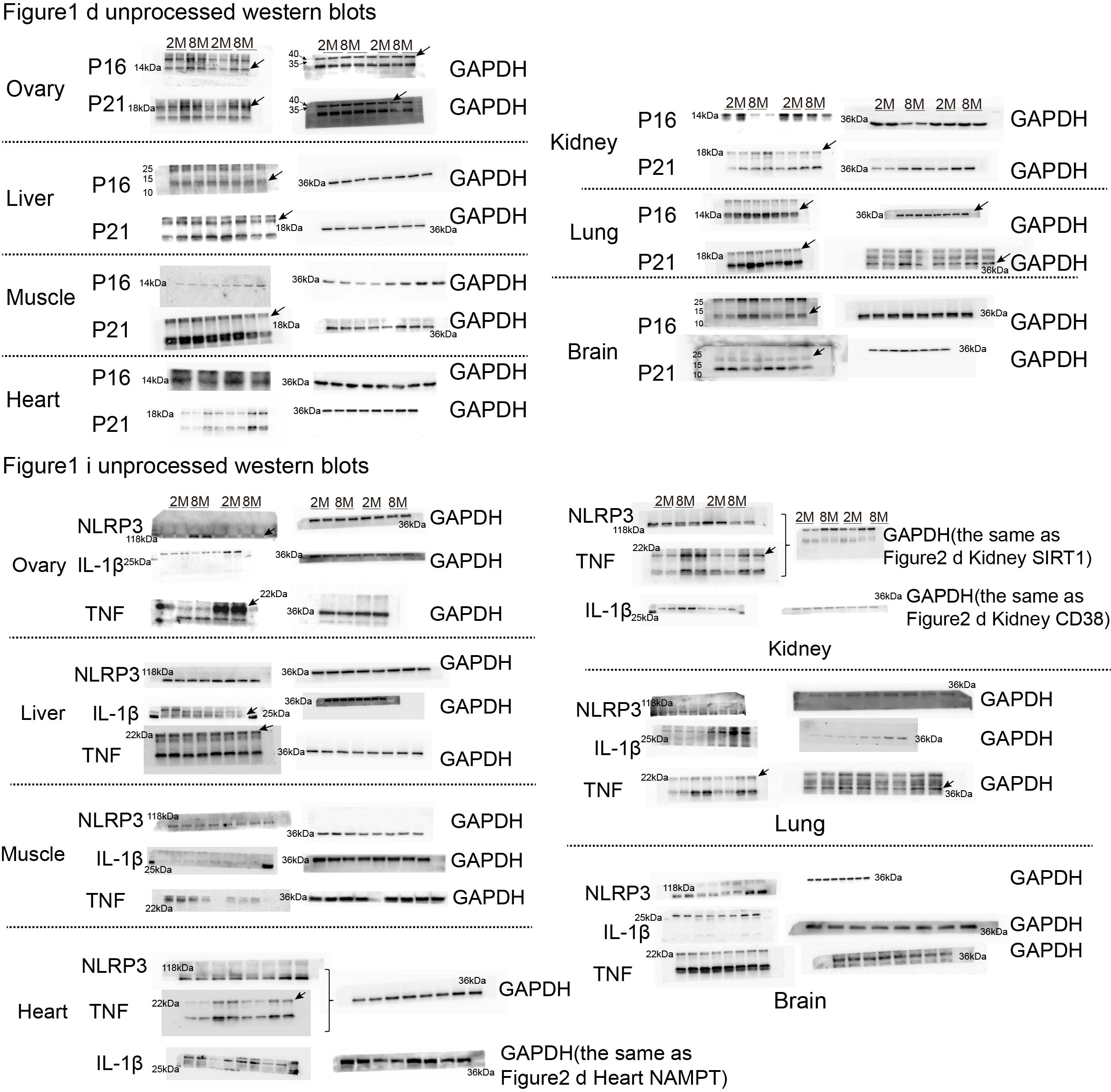

Supplement: Supplementary file 5 — Unprocessed western blots. [file 43587_2023_532_MOESM5_ESM.jpg]

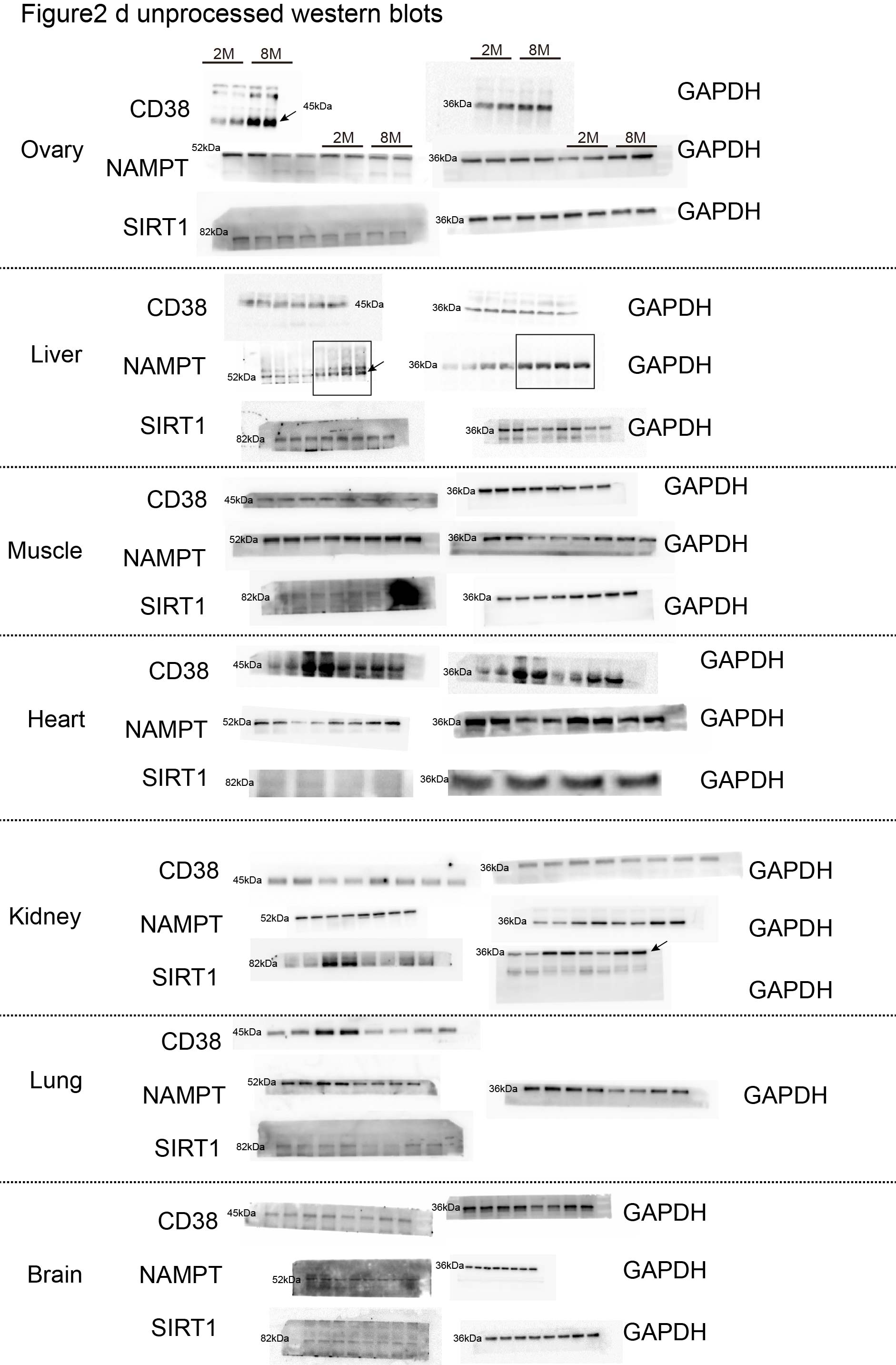

Supplement: Supplementary file 7 — Unprocessed western blots. [file 43587_2023_532_MOESM7_ESM.jpg]

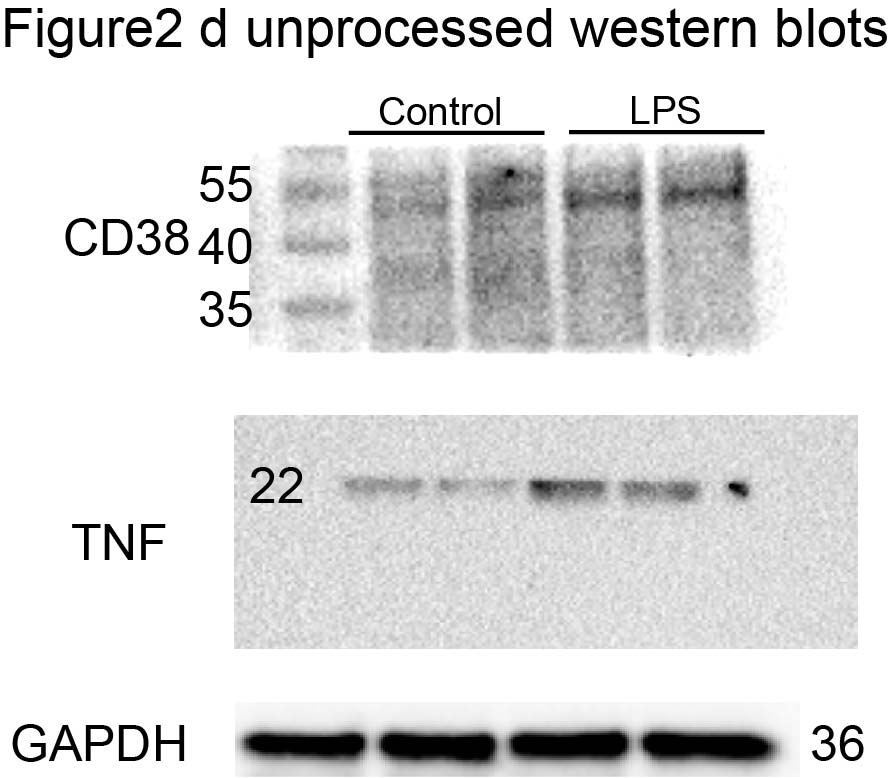

Supplement: Supplementary file 11 — Unprocessed western blots. [file 43587_2023_532_MOESM11_ESM.jpg]

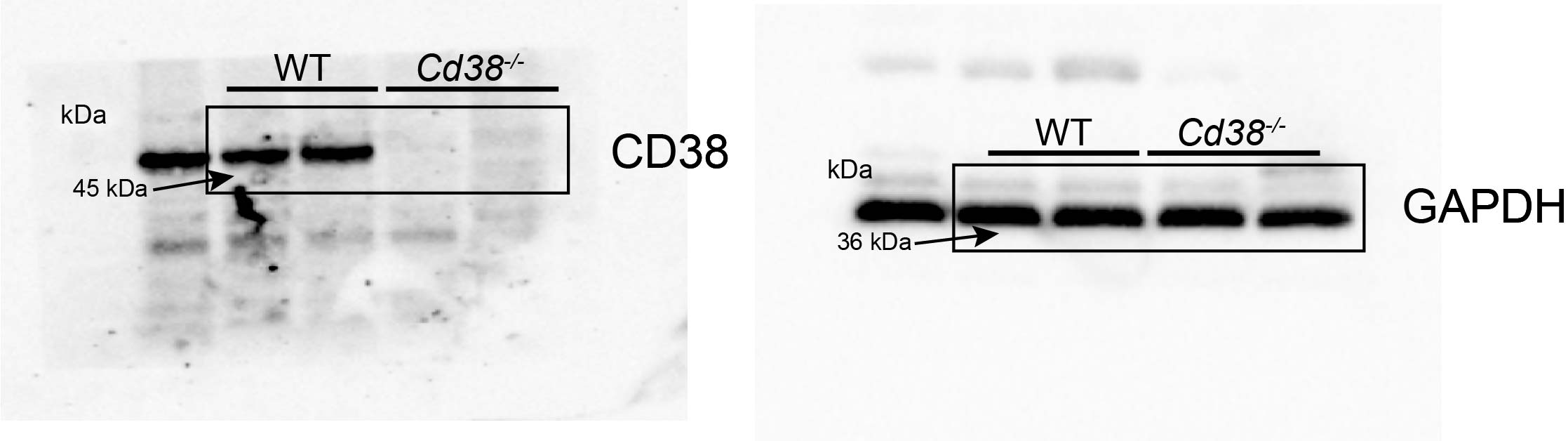

Supplement: Supplementary file 14 — Unprocessed western blots. [file 43587_2023_532_MOESM14_ESM.jpg]
